# β-Arrestin-1 is required for adaptive β-cell mass expansion during obesity

**DOI:** 10.1038/s41467-021-23656-1

**Published:** 2021-06-07

**Authors:** Luiz F. Barella, Mario Rossi, Sai P. Pydi, Jaroslawna Meister, Shanu Jain, Yinghong Cui, Oksana Gavrilova, Gianluca Fulgenzi, Lino Tessarollo, Jürgen Wess

**Affiliations:** 1 Molecular Signaling Section, Laboratory of Bioorganic Chemistry, National Institute of Diabetes and Digestive and Kidney Diseases, Bethesda, MD USA; 2 Molecular Recognition Section, Laboratory of Bioorganic Chemistry, National Institute of Diabetes and Digestive and Kidney Diseases, Bethesda, MD USA; 3 Mouse Metabolism Core, National Institute of Diabetes and Digestive and Kidney Diseases, Bethesda, Bethesda, MD USA; 4 Mouse Cancer Genetics Program, National Cancer Institute, Frederick, MD USA

**Keywords:** Diabetes, Type 2 diabetes, Preclinical research

## Abstract

Obesity is the key driver of peripheral insulin resistance, one of the key features of type 2 diabetes (T2D). In insulin-resistant individuals, the expansion of beta-cell mass is able to delay or even prevent the onset of overt T2D. Here, we report that beta-arrestin-1 (barr1), an intracellular protein known to regulate signaling through G protein-coupled receptors, is essential for beta-cell replication and function in insulin-resistant mice maintained on an obesogenic diet. Specifically, insulin-resistant beta-cell-specific *barr1* knockout mice display marked reductions in beta-cell mass and the rate of beta-cell proliferation, associated with pronounced impairments in glucose homeostasis. Mechanistic studies suggest that the observed metabolic deficits are due to reduced Pdx1 expression levels caused by beta-cell barr1 deficiency. These findings indicate that strategies aimed at enhancing barr1 activity and/or expression in beta-cells may prove useful to restore proper glucose homeostasis in T2D.

## Abstract (web summary)

During insulin-resistance, the compensatory expansion of beta-cell mass is able to delay or the onset of overt type 2 diabetes. Here, the authors report that beta-arrestin-1, an intracellular protein known to regulate signalling through G protein-coupled receptors, is essential for beta-cell replication and function in insulin-resistant mice.

## Introduction

In obese individuals, peripheral tissues are impaired in their ability to properly respond to insulin, leading to the development of insulin resistance, a hallmark of type 2 diabetes (T2D)^1–3^. Initially, insulin resistance leads to a compensatory increase in beta-cell mass that can delay or even prevent the development of T2D^4–7^_._

During the past decade, several factors have been identified that promote beta-cell hypertrophy and replication in insulin-resistant states^4–6^. However, our knowledge about the intracellular signaling pathways that modulate these adaptive changes in beta-cell mass remains incomplete. Clearly, the identification of additional signaling molecules that are able to promote beta-cell proliferation may lead to improved therapies to prevent the progression of peripheral insulin resistance to T2D.

We and others recently demonstrated that two intracellular signaling molecules, beta-arrestin-1 and -2 (barr1 and barr2, respectively), regulate the activity of many metabolically important cell types including pancreatic beta-cells^8–11^. These two proteins are well known for their ability to dampen signaling through G protein-coupled receptors (GPCRs)^12^. In addition, beta-arrestins can also act as signaling molecules in their own right^13,14^. For example, it has been demonstrated that barr1 is present in the cell nucleus where it can promote the transcription of various genes^15–18^.

The two beta-arrestins are present in virtually all cell types^19^. Both barr1 and barr2 are already expressed during early development^19,20^. RNA-seq studies have shown that *barr1* and *barr2* are expressed in both mouse^21^ and human^22^ beta-cells, but that *barr1* RNA is at least tenfold less abundant than *barr2* RNA in mouse beta-cells. We recently demonstrated that mutant mice selectively lacking barr2 in pancreatic beta-cells show striking metabolic deficits, including impaired insulin secretion and reduced glucose tolerance, and that barr2 represents a key regulator of the activity of CAMKII in beta-cells^10^. Although both beta-arrestins interfere with GPCR signaling, accumulating evidence suggests that barr1 and barr2 have distinct functional roles and can even have antagonistic effects^23^. For this reason, we decided to use gene targeting technology to explore the potential role of beta-cell barr1 on beta-cell function and whole body glucose homeostasis.

We previously reported that beta-cell-specific *barr1* knockout mice (beta-barr1-KO mice) consuming a standard chow did not show any significant deficits in glucose homeostasis including glucose-stimulated insulin secretion (GSIS) and insulin tolerance^24^. However, we found that the ability of certain sulfonylurea drugs to stimulate insulin secretion with high efficacy was dependent on the presence of beta-cell barr1^24^. Mechanistic data showed that this phenotype was due to the ability of barr1 to facilitate sulfonylurea-induced insulin release via Epac2, which requires the formation of a barr1/Epac2 complex for full functional activity^24^. However, the activity of other insulin secretagogues remained unaffected in beta-barr1-KO mice maintained on standard chow.

The present study was designed to examine whether barr1 was required for the proper function of beta-cells under metabolically challenging conditions such as impaired peripheral insulin resistance caused by an obesogenic diet. We show that insulin-resistant beta-barr1-KO mice display pronounced reductions in beta-cell mass and rate of beta-cell proliferation, leading to severely impaired glucose homeostasis. Using a combination of different experimental approaches and tools, including human EndoC-βH1 beta-cells, we provide evidence that the lack of beta-cell barr1 leads to reduced expression of Pdx1, the master regulator of beta-cell function and beta-cell mass expansion^25,26^. We also show that beta-cell barr1 deficiency causes decreased Pdx1 expression levels and that reduced Pdx1 expression is central to the metabolic phenotypes displayed by the barr1 mutant mice. These findings suggest that approaches aimed at enhancing the activity or expression levels of beta-cell barr1 may prove useful to increase beta-cell mass and function for the treatment of T2D.

## Results

### Generation of beta-cell-selective *barr1* knockout mice

We inactivated the *barr1* (*arrb1*) gene in beta-cells of adult mice^24^. To generate the experimental animals used in the present study, we crossed floxed *barr1* mice (*fl/fl barr1* mice)^27^ with *Pdx1-Cre-ER*^*TM*^ transgenic mice^28,29^. Tamoxifen treatment of *fl/fl barr1*-*Pdx1-Cre-ER*^*TM*^ mice resulted in beta-barr1-KO mice that selectively lacked barr1 in beta-cells^24^. *Barr1* expression levels remained unaffected in other metabolically important tissues including the hypothalamus^24^. Tamoxifen-treated floxed *barr1* mice (*fl/fl barr1* littermates) served as control animals throughout all experiments. Previous work has shown that female C57BL/6 mice are protected against the detrimental metabolic effects caused by the consumption of a high-fat diet (HFD), including insulin resistance and glucose intolerance^30,31^. For this reason, only male mice were analyzed in the present study (genetic background: C57BL/6NTac).

### In vivo metabolic studies with obese beta-barr1-KO mice

**Fig. 1:**
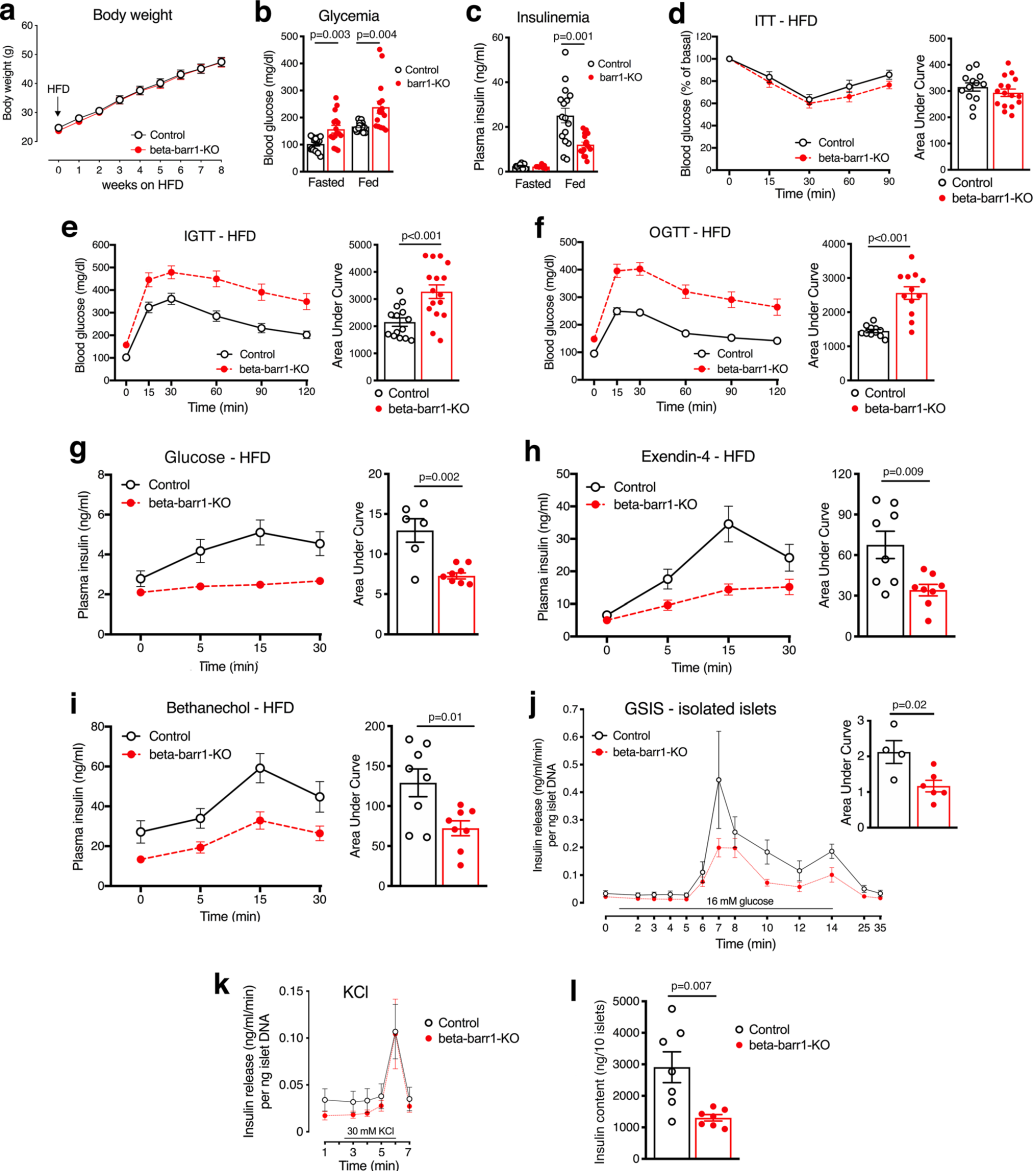
Selective deletion of barr1 in beta-cells greatly impairs glucose homeostasis and stimulated insulin release in obese mice. For all experiments, adult male mice (beta-barr1-KO mice and control littermates) maintained on a high-fat diet (HFD) for at least 8 weeks were used. **a** Body weight measurements. **b**, **c** Blood glucose (**b**) and plasma insulin (**c**) levels in freely fed or fasted (14–16 h overnight) mice. **d** Insulin tolerance test (ITT). Mice were fasted for 4 h and then injected with insulin (1 U/kg i.p.). **e** I.p. glucose tolerance test (IGTT). After an overnight fast, mice were injected with glucose (1 g/kg i.p.). **f** Oral glucose tolerance test (OGTT). After an overnight fast, mice received glucose via oral gavage (1 g/kg i.p.). **g** Glucose-stimulated plasma insulin levels in vivo. Mice that had been fasted overnight were injected with glucose (1 g/kg i.p.), followed by the monitoring of plasma insulin levels. **h** Exendin-4-stimulated insulin secretion in vivo. Mice that had been fasted for 4 h were injected with exendin-4 (12 nmoles/kg i.p.) and glucose (1 g/kg i.p.). **i** Bethanechol-stimulated insulin secretion in vivo. Freely fed mice were injected with bethanechol (2 mg/kg s.c.). **j** GSIS is impaired in perifused islets from beta-barr1-KO mice. Pancreatic islets prepared from HFD control and beta-barr1-KO mice were perifused with 16 mM glucose, as indicated. The amount of secreted insulin was normalized to islet DNA content. (*n* = 4–6 perifusions with 50 islets per perifusion chamber; islets were isolated from five mice per genotype). **k** KCl-induced insulin secretion remains unaffected by beta-cell barr1 deficiency. KCl (30 mM) was added at the end of each perifusion experiment (see (**j**) for details). **l** Insulin content is reduced in islets from HFD beta-barr1-KO mice (seven separate batches of ten islets from at least three different mice per genotype were analyzed). Data shown were means ± s.e.m. (number of mice per group: **a** and **b**, 14 control and 16 KO mice, respectively, **c**, 8 fasted and 16 fed mice, respectively; **d**, 13 control and 16 KO mice, respectively; **e**, 14 control and 16 KO mice, respectively; **f**, 11 control and 12 KO mice, respectively; **g**, 7; **h**, 8; **i**, 8) (mouse age: 17–24 weeks). *P* values are indicated in the different panels (unpaired two-tailed *t*-test). Source data are provided as a Source data file.

Our goal was to investigate whether barr1 is required for the proper function of beta-cells under metabolically challenging conditions such as impaired peripheral insulin resistance caused by an obesogenic diet. To address this question, we maintained beta-barr1-KO mice and control littermates on a HFD for at least 8 weeks (HFD feeding was initiated 2 weeks after the last tamoxifen injection). Consumption of the HFD resulted in comparable increases in body weight in the mutant and control mice (Fig. 1a). Strikingly, however, beta-barr1-KO mice exhibited a pronounced increase in blood glucose levels under both fed and fasting conditions and a significant reduction in plasma insulin levels in the fed state, as compared with their control littermates (Fig. 1b, c). The two groups of mice showed similar reductions in blood glucose levels in an insulin tolerance test (ITT; Fig. 1d). In contrast, the HFD beta-barr1-KO mice showed a severe impairment in glucose tolerance, as shown in i.p. and oral glucose tolerance tests (Fig. 1e, f).

On the basis of these observations, we examined whether the lack of beta-cell barr1 led to impaired insulin secretion in obese beta-barr1-KO mice. We found that glucose-stimulated increases in plasma insulin levels (1 g glucose/kg i.p.) were profoundly reduced in the HFD beta-barr1-KO mice (Fig. 1g). Glucose-induced insulin release is modulated by the activity of various GPCRs, including beta-cell M3 muscarinic^32^ and beta-cell GLP-1 receptors^33,34^. To examine whether the increase in insulin release caused by agonist activation of these receptors was also affected by beta-cell barr1 deficiency, we injected HFD control and beta-barr1-KO mice with bethanechol (2 mg/kg s.c.), a muscarinic receptor agonist, or exendin-4 (12 nmol/kg i.p.), a GLP-1 receptor agonist. As observed with glucose-injected mice, treatment with either exendin-4 (Fig. 1h) or bethanechol (Fig. 1i) caused significantly reduced insulin responses in beta-barr1-KO mice, as compared to control littermates.

Since blood glucose and plasma insulin levels were altered in HFD beta-barr1-KO mice, we also investigated whether plasma glucagon levels were affected by beta-cell barr1 deficiency. We measured plasma glucagon levels in fasted (14–16 h overnight) and freely fed HFD beta-barr1-KO and control mice. We found that plasma glucagon levels remained unaffected by the lack of beta-cell barr1 (Supplementary Fig. 1).

### Studies with isolated pancreatic islets prepared from obese beta-barr1-KO mice

To confirm that the impairment in glucose-induced increases in plasma insulin levels caused by beta-cell barr1 deficiency in vivo was caused by deficient beta-cell function, we carried out a series of in vitro studies using perifused islets prepared from HFD beta-barr1-KO mice and control littermates. In agreement with the in vivo data, the in vitro experiments showed that glucose (16 mM)-stimulated insulin secretion was severely reduced in the perifused mutant islets (Fig. 1j). In contrast, KCl (30 mM)-induced insulin secretion remained unaffected by beta-cell barr1 deficiency (Fig. 1k). Additional studies showed that pancreatic islets prepared from HFD beta-barr1-KO mice showed ~50% reduction in insulin content (Fig. 1l), providing a likely explanation for the reduction in insulin release observed with the mutant islets.

**Fig. 2:**
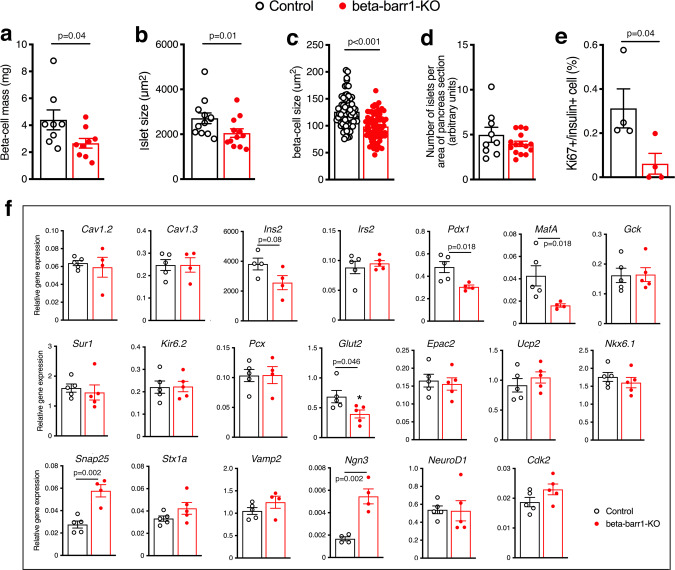
Islets from HFD beta-barr1-KO mice show deficits in beta-cell mass, size, and proliferation rate, and altered expression of key beta-cell genes. **a–c** Beta-cell mass (**a**), islet size (**b**), and beta-cell size (**c**) are significantly decreased in HFD beta-barr1-KO mice, as compared to HFD control mice. **d** Number of islets per area of pancreas section (arbitrary units). **e** Rate of beta-cell proliferation (Ki67/insulin co-staining experiments). **f** Expression levels of genes involved in the maintenance and function of beta-cells, as studied by qRT-PCR. RNA was isolated from pancreatic islet from HFD control and beta-barr1-KO mice. Data were given as means ± s.e.m. (**a**, 8 control and 9 KO mice, respectively; **b**, 9 control and 15 KO mice, respectively; **c**, 75 and 73 cells, respectively, were examined from three mice per group; **d**, 9 control and 15 KO pancreatic sections were examined from three mice per group; **e**, data were from four mice per genotype; **f**, data were from at least four mice per genotype). *P* values are indicated in the different panels (unpaired two-tailed *t*-test). Source data are provided as a Source data file.

Given the pronounced reduction in islet insulin content caused by beta-cell barr1 deficiency (Fig. 1l), we next carried out a series of morphometric studies using pancreatic slices prepared from HFD beta-barr1-KO mice and control littermates. Interestingly, beta-barr1-KO islets exhibited a significant decrease in beta-cell mass, and mean islet and beta-cell size (Fig. 2a–c). However, islet density was similar in beta-barr1-KO and control islets (Fig. 2d). Similarly, alpha-cell and delta-cell mass remained unchanged by the lack of beta-cell barr1 (Supplementary Fig. 2), and the relative spatial distribution of alpha- versus beta-cells was similar in mutant and control islets (Supplementary Fig. 3).

### Beta-cell proliferation is greatly reduced in HFD beta-barr1-KO islets

The consumption of an obesogenic diet stimulates beta-cell replication to promote an increase in beta-cell mass required to maintain euglycemia despite peripheral insulin resistance^4–6,35^. This increase in beta-cell mass is achieved by beta-cell hypertrophy, combined with an increase in beta-cell proliferation^4–6,35^. Prompted by these findings, we double-stained pancreatic slices for insulin and Ki67, a marker of beta-cell proliferation^35^. Strikingly, we found that beta-cell proliferation was nearly abolished in HFD beta-barr1-KO islets (Fig. 2e). In contrast, a significant percentage of control beta-cells were Ki67-positive under the same experimental conditions (Fig. 2e). Using a TUNEL assay, we did not detect any apoptotic beta-cells in pancreatic slices from either HFD control or HFD beta-barr1-KO pancreata (*n* = 5 mice/pancreata per genotype). Total pancreatic weight did not differ significantly between HFD beta-barr1-KO and control mice (control: 375 ± 38 mg; KO: 333 ± 11 mg; *n* = 3 per genotype).

### Electron microscopy studies

Electron microscopy studies confirmed that islets/beta-cells from HFD beta-barr1-KO mice did not exhibit any significant structural changes, as compared to HFD control mice (Supplementary Fig. 4a). However, the mutant islets showed a significant decrease in the number of insulin granules pre-docked to the beta-cell plasma membrane (also known as readily releasable insulin pool; Supplementary Fig. 4a, b). This observation is consistent with the observation that the initial glucose-induced peak in insulin release was blunted in perifused islets lacking barr1 in beta-cells (Fig. 1j). The number of total granules per μm^2^ of beta-cell area was similar in control and mutant islets (Supplementary Fig. 4c).

We also found that the number of “immature/empty” granules was similar in both groups (~5%). Since insulin content was decreased in islets from HFD beta-barr1-KO mice, it is likely that the decrease in mean islet and beta-cell size is responsible for this phenotype (Fig. 2b, c).

### Beta-cell barr1 deficiency leads to reduced Pdx1 expression and alters the expression of several other important genes

**Fig. 3:**
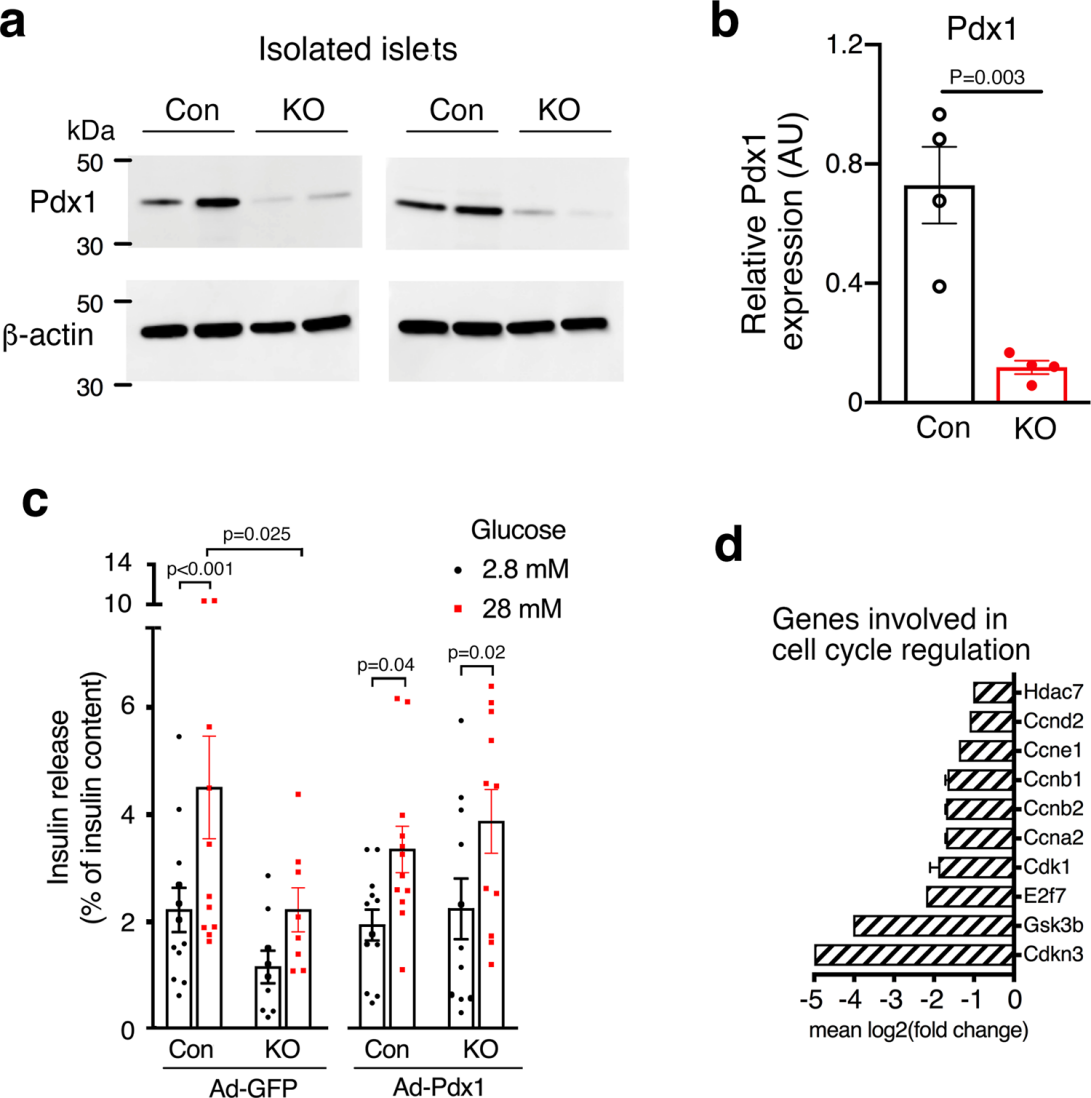
Reduced Pdx1 expression in HFD beta-barr1-KO islets, Pdx1 rescue experiments, and reduced expression of cell cycle genes. **a** Reduced expression of Pdx1 protein in the absence of beta-cell barr1. Lysates from pancreatic islets derived from HFD control (Con) and beta-barr1-KO (KO) mice were subjected to Western blotting studies (see Methods for details). Blots were probed with anti-Pdx1 and anti-β-actin antibodies. **b** Quantification of the Western blotting data shown in (**a**) (*n* = 4 per genotype). Pdx1 protein expression was normalized to the expression of β-actin. **c** Overexpression of Pdx1 in HFD beta-barr1-KO islets restores normal GSIS. Islets from HFD control and beta-barr1-KO mice were infected with either a control adenovirus (Ad-GFP) or an adenovirus coding for Pdx1 (Ad-Pdx1). GSIS was studied in static islet incubation assays. Islets were incubated for 1 h in Krebs solution containing 2.8 or 28 mM glucose. The amount of insulin secreted into the medium was normalized to the total insulin content of the islets in each well. Most notably, mutant islets treated with Ad-Pdx1 regained a similar degree of GSIS as Ad-Pdx1- or Ad-GFP-treated control islets (8–12 islet preparations from four mice per genotype). **d** Reduced expression of key cell cycle genes in HFD beta-barr1-KO islets, as determined by RNA-seq. See Methods for experimental details (mouse age: ~20 weeks; *n* = 6 mice/genotype). *P* values are indicated in the different panels (**b**: unpaired two-tailed *t*-test; **c**: two-way Anova followed by Sidak’s post hoc test). Source data are provided as a Source data file.

Beta-cell function and replication are controlled by a large number of genes. Initially, we used qRT-PCR to study whether beta-cell barr1 deficiency affected the expression of key genes involved in beta-cell function and replication, using islet RNA prepared from HFD beta-barr1-KO mice and control littermates as a template. This analysis identified several genes that showed decreased expression levels in the mutant islets including *Pdx1*, *MafA*, and *Glut2* (Fig. 2f). The expression of *Ins2* was also reduced, although this effect failed to reach statistical significance (Fig. 2f). Western blotting studies confirmed that the expression of Pdx1 protein was also drastically reduced in islets from HFD beta-barr1-KO mice (Fig. 3a, b). We also noted that the expression of *Ngn3* and *Snap25* was significantly increased in islets prepared from HFD beta-barr1-KO mice (Fig. 2f).

### Adenovirus-mediated expression of Pdx1 in beta-barr1-KO islets restores normal GSIS

The observed reduction of Pdx1 expression in the beta-barr1-KO islets was of particular interest (Fig. 3a, b). Pdx1 is considered the key transcription factor promoting beta-cell differentiation and replication and ensuring proper beta-cell function^36–38^. Moreover, Pdx1, in concert with other transcription factors, strongly activates the expression of several other functionally critical beta-cell genes including *Ins2, MafA*, and *Glut2*^39–42^ and is required for the increase in beta-cell mass caused by peripheral insulin resistance^43,44^.

On the basis of these findings, we speculated that reduced Pdx1 expression is the primary cause of the impaired beta-cell function observed with HFD beta-barr1-KO islets (Fig. 1j). To test this hypothesis in a more direct fashion, we infected islets from HFD control and beta-barr1 mice with either a control adenovirus (Ad-GFP) or an adenovirus coding for Pdx1 (Ad-Pdx1). We then studied GSIS in static islet incubation assays. As expected from the outcome of the islet perifusion studies (Fig. 1j), GSIS was significantly reduced in the mutant islets treated with the control virus (Ad-GFP), as compared to Ad-GFP-treated control islets (Fig. 3c). Strikingly, mutant islets treated with Ad-Pdx1 regained a similar degree of GSIS as Ad-Pdx1- or Ad-GFP-treated control islets (Fig. 3c). This rescue experiment strongly supports the concept that Pdx1 deficiency resulting from the lack of beta-cell barr1 is the primary cause of impaired beta-cell function displayed by beta-barr1-KO islets.

### RNA-seq studies

To examine gene expression in a more general fashion, we performed RNA-seq studies using RNA prepared from islets of HFD control and beta-barr1-KO mice as a template. Given a cut-off of log_2_(1) or twofold, 263 genes were upregulated, whereas 841 genes were downregulated in the mutant islets. A summary of the most significantly enriched signaling pathways for significantly downregulated genes is shown in Supplementary Fig. 5a. Interestingly, several of these pathways involved genes critical for cell cycle regulation, including genes coding for various cyclins and cyclin-dependent kinases (see Supplementary Fig. 5b for a Volcano plot highlighting key genes). Heat maps showing the differential expression of genes involved in cell cycle (GO term: 0007049), cyclin-dependent protein kinase activity (GO term: 0097472), and glucose homeostasis (GO term: 0042593) are shown in Supplementary Fig. 6.

Figure 3d highlights key cyclin genes (*Ccnd2, Ccne1*, *Ccnb1, Ccnb2*, and *Ccna2)* and several other genes involved in cell cycle progression (*Hdac7*, *Cdk1*, and *E2f7*) that were strongly downregulated in islets lacking barr1 in beta-cells. Since many of these genes are critically involved in beta-cell replication^45,46^, our RNA-seq data are consistent with a key role of barr1 in promoting beta-cell proliferation.

### Studies with cultured mouse and human beta-cell lines

**Fig. 4:**
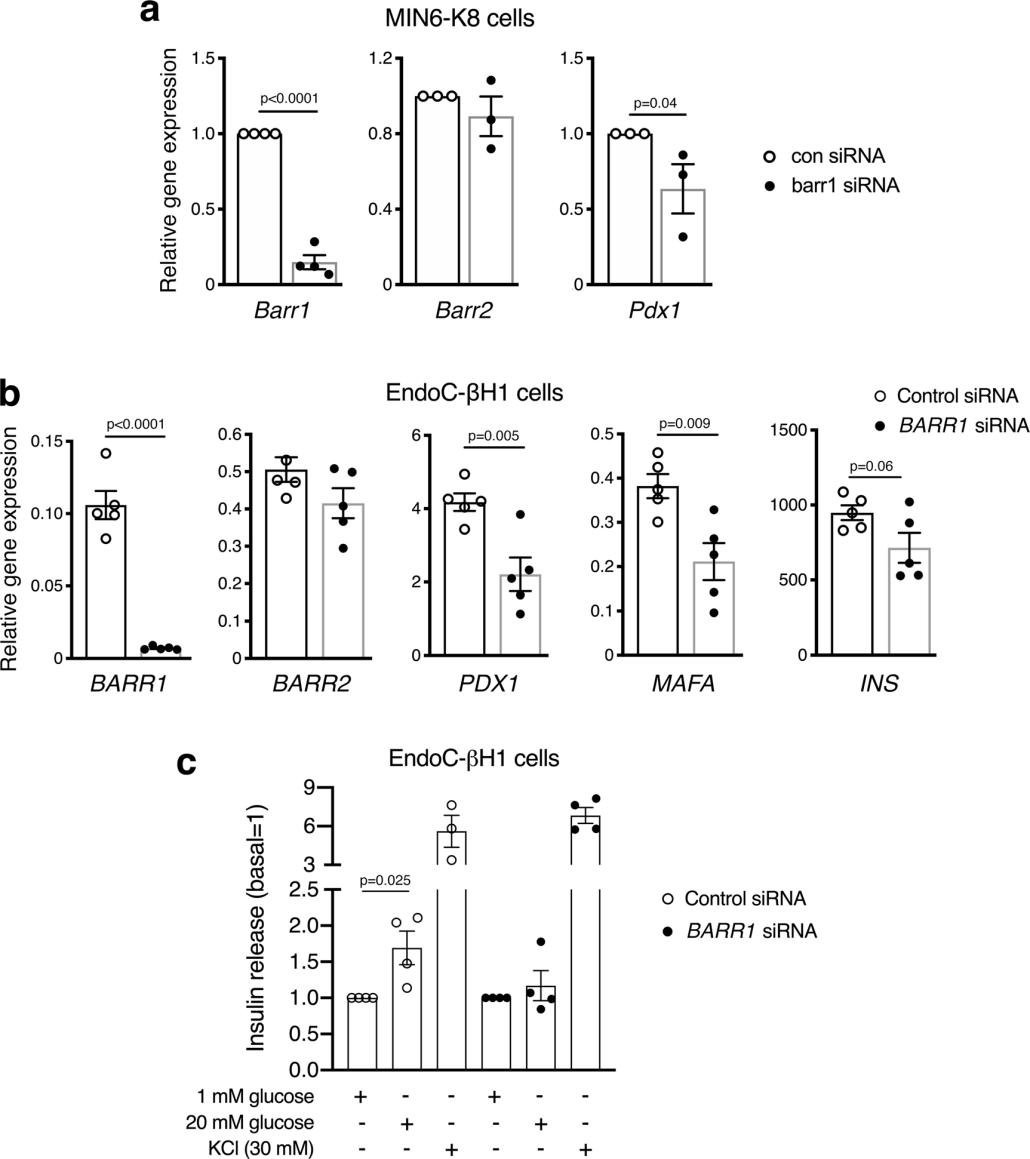
Gene expression and functional studies with barr1-deficient mouse and human beta-cells. **a** siRNA-mediated knockdown of *barr1* expression in mouse MIN6-K8 cells. *Pdx1* gene expression was significantly decreased after *barr1* knockdown, while *barr2* expression remained unchanged. Gene expression data were normalized relative to the expression of *β-actin*. **b** siRNA-mediated knockdown of *BARR1* expression in human EndoC-βH1 cells. While *BARR2* expression remained unchanged, *PDX1, MAFA*, and *INS* transcript levels were decreased after *BARR1* knockdown. Gene expression data were normalized relative to the expression of *HPRT*. **c** Knockdown of *BARR1* expression in EndoC-βH1 cells strongly impairs GSIS. Insulin release assays were carried out with EndoC-βH1 cells that had been treated with *BARR1* or control (scrambled) siRNA. Cells were incubated with 1 mM glucose, 20 mM glucose, or 30 mM KCl. Insulin secretion was normalized to basal values obtained at 1 mM glucose (=1). Data were given as means ± s.e.m. from four independent experiments. *P* values are indicated in the different panels (unpaired two-tailed *t*-test). Source data are provided as a Source data file.

To further confirm the link between reduced *barr1* and *Pdx1* expression, we used cultured MIN6-K8 mouse insulinoma cells^47^ as an in vitro model system. We found that siRNA-mediated knockdown of *barr1* expression had no significant effect on *barr2* transcript levels but led to a ~50% reduction in *Pdx1* gene expression (Fig. 4a). This observation is in good agreement with the data obtained with isolated islets from beta-barr1-KO mice (Figs. 2f and 3a, b).

We next wanted to investigate whether barr1 also regulates the expression of *PDX1* in human beta-cells. To address this question, we used human EndoC-βH1 beta-cells which closely mimic the functional properties of native human beta-cells^48,49^. qRT-PCR studies demonstrated that treatment of EndoC-βH1cells with *BARR1* siRNA resulted in a ~80–90% reduction in *BARR1* gene expression levels (Fig. 4b). Moreover, in agreement with the results obtained with mouse beta-cells, knockdown of *BARR1* also resulted in a significant reduction of *PDX1* expression in EndoC-βH1 cells (Fig. 4b)*. BARR1* knockdown also decreased the expression of *MAFA (P* < *0.01)* and *INS* (*P* = 0.06), while *BARR2* mRNA levels remained unaffected (Fig. 4b).

Treatment of EndoC-βH1 cells with a high concentration of glucose (20 mM) led to a significant increase in insulin secretion (Fig. 4c). Strikingly, this response was completely absent in EndoC-βH1 cells treated with *BARR1* siRNA (Fig. 4c). In contrast, KCl (30 mM)-induced insulin secretion remained unaffected by *BARR1* knockdown (Fig. 4c), suggesting that BARR1 deficiency does not affect distal events (e.g., the exocytosis machinery) involved in insulin secretion. Insulin content remained unaffected by *BARR1* knockdown (normalized total insulin content in mU/l: control siRNA treatment, 73.8 ± 21.9; *BARR1* siRNA treatment, 69.2 ± 18.0; means ± s.e.m.; *n* = 4 per group).

**Fig. 5:**
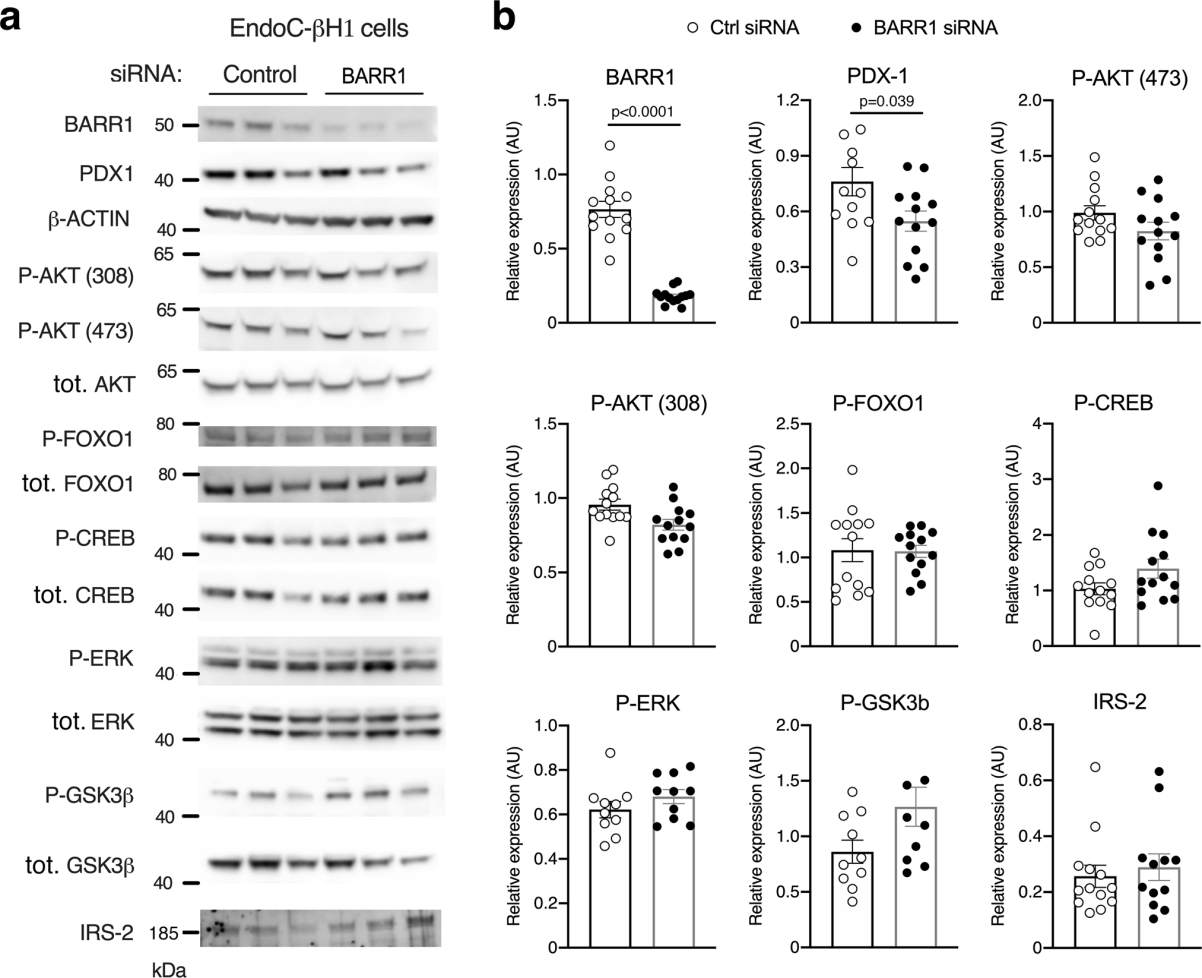
Effect of knockdown of *BARR1* in EndoC-βH1 cells on protein expression levels of key signaling proteins. **a** Lysates from EndoC-βH1 cells that had been treated with control or *BARR1* siRNA were subjected to Western blotting studies. Blots were probed with the indicated antibodies. **b** Quantification of the Western blotting data shown in (**a**). Protein expression was normalized to β-actin or, in the case of phospho-proteins, to the corresponding total protein expression levels. Data were given as means ± s.e.m. from four independent experiments. *P* values are indicated in panel **b** (unpaired two-tailed *t*-test). Source data are provided as a Source data file.

In agreement with the qRT-PCR studies, Western blotting studies showed that treatment of EndoC-βH1cells with *BARR1* siRNA led to a pronounced reduction in BARR1 expression and a significant decrease in PDX1 protein expression levels, as compared to cells treated with scrambled control siRNA (Fig. 5). On the other hand, the expression levels of AKT, FOXO1, CREB, ERK, GSK3β, and IRS2, as well as the expression levels of the phosphorylated forms of these signaling proteins/transcription factors (p-AKT, p-FOXO1, p-CREB, p-ERK, and p-GSK3β), remained essentially unchanged after *BARR1* knockdown (Fig. 5).

Since barr1 can regulate transcriptional processes in the nucleus^15–18^, we studied the cellular localization of BARR1 in EndoC-βH1 cells. Using a standard cell fractionation protocol, we prepared nuclear and cytosolic protein extracts, followed by Western blotting studies. We found that BARR1 was present both in the nucleus and cytoplasm (Supplementary Fig. 7). Treatment of EndoC-βH1 cells with *BARR1* siRNA significantly decreased BARR1 expression in both fractions (Supplementary Fig. 7).

### Transcriptional regulation of *Pdx1* expression by barr1

Several studies have shown that nuclear barr1 is able to alter gene expression at the transcriptional level^15–18^. We therefore tested the hypothesis that barr1 might be required for the efficient transcriptional activation of the *Pdx1* promoter. Specifically, we transiently transfected MIN6-K8 cells with a reporter construct (Pdx1-pGL3)^50^ containing the luciferase gene under the transcriptional control of the mouse *Pdx1* promoter or with empty vector (pGL3). The pGL3-Pdx1 plasmid harbors a 1927-bp fragment containing 1886 bp upstream and 41 bp downstream of the transcriptional start site of the mouse *Pdx1* promoter. As reported previously^50^, this *Pdx1* promoter sequence is endowed with strong transcriptional activity containing binding sites for multiple transcription factors including GATA4/GATA6^50^, products of different MODY genes^51^, Ptf1a^52^, and HNF-6^53^. Moreover, promoter analysis software (AliBaba2.1) predicts the presence of many additional transcription factor binding sites within the *Pdx1* promoter sequence contained in the luciferase reporter construct.

**Fig. 6:**
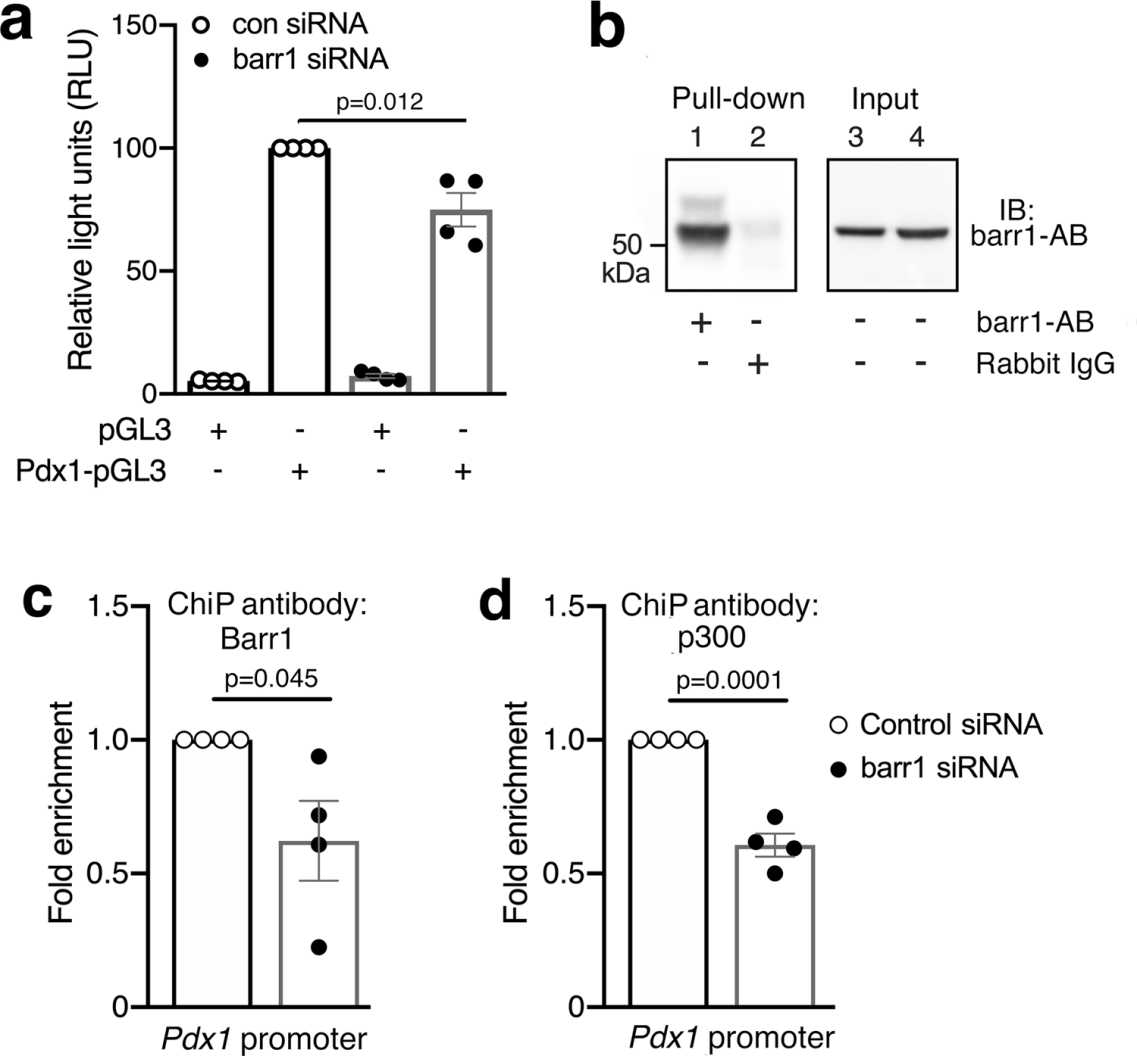
*Pdx1* promoter and ChIP experiments performed with MIN6-K8 cells. **a** Activation of a *Pdx1* promoter-luciferase construct (*Pdx1-pGL3*) in MIN6-K8 cells is significantly reduced after *barr1* knockdown. *pGL3*: empty vector (negative control). **b** Representative pull-down assay demonstrating the selectivity of the barr1 antibody used for ChIP experiments. Lysates from MIN6-K8 cells were incubated with Sepharose beads coated with barr1 antibody or normal rabbit IgG, followed by elution of captured proteins and Western blotting studies. **c**, **d** ChIP experiments were carried with MIN6-K8 cells transfected with either scrambled control or *barr1* siRNA, using antibodies against barr1 (**c**) or p300 (**d**). The presence of specific *Pdx1* promoter sequences in the input DNA recovered from the antibody-bound chromatin segments were analyzed by qPCR (see text for details). Data were normalized to the corresponding input controls. Data were given as means ± s.e.m. from four independent experiments. *P* values are indicated in the different panels (unpaired two-tailed *t*-test). Source data are provided as a Source data file.

Expectedly, MIN6-K8 cells treated with the empty vector alone showed only residual luciferase activity (Fig. 6a). In contrast, MIN6-K8 cells transfected with control siRNA and the Pdx1-pGL3 plasmid displayed a pronounced increase in luciferase activity (Fig. 6a). This response was significantly reduced after siRNA-mediated knockdown of *barr1* expression (Fig. 6a), indicating that the presence of barr1 is required for efficient *Pdx1* transcription.

It has been shown that p300, a histone acetyltransferase, is critical for the efficient activation of the *Pd1x* promoter^54^. Moreover, Kang et al.^15^ demonstrated that the binding of nuclear barr1 to p300 can enhance the transcription of various genes. For these reasons, we performed chromatin immunoprecipitation (ChIP) experiments, using ChIP primers that amplified regions in the *Pdx1* promoter containing potential binding sites for p300-dependent transcriptional complexes^55^. Specifically, we prepared nuclear DNA from MIN6-K8 cells transfected with either control or *barr1* siRNA. A pull-down assay confirmed that the barr1 antibody used for the ChIP experiments selectively immunoprecipitated barr1 protein (Fig. 6b), in agreement with data provided by the manufacturer (Cell Signaling). Interestingly, *barr1* knockdown led to a significant decrease in fold enrichment of a *Pdx1* promoter sequence (nucleotides −1090 to −976 relative to the transcription start site) in input DNA recovered from barr1 antibody-bound chromatin segments (Fig. 6c). Promoter analysis software (AliBaba2.1) predicts that this region contains binding sites for multiple transcription factors including Sp1, SRF, and Oct1. We obtained similar results with the p300 antibody which protected the same promoter region as the barr1 antibody (Fig. 6d). These observations strongly suggest that nuclear barr1 plays a key role in regulating *Pdx1* gene expression and that barr1 most likely forms a complex with p300 and other nuclear factors to promote *Pdx1* transcription.

ChIP experiments also showed that knockdown of *barr1* in MIN6-K8 cells caused a significant decrease in fold enrichment of *Mafa* and *Cdkn3* promoter sequences in input DNA recovered from barr1 antibody- or p300 antibody-bound chromatin segments (Supplementary Fig. 8a–d). The protected promoter sequences comprised nucleotides −380 to −230 and −760 to −635, respectively (transcription start site: +1). Both regions are predicted to harbor potential binding sites for p300-dependent transcriptional complexes^55^. These findings suggest that nuclear barr1 probably also regulates the expression of other important beta-cell genes.

### Metabolic studies with transgenic mice selectively overexpressing barr1 in beta-cells

**Fig. 7:**
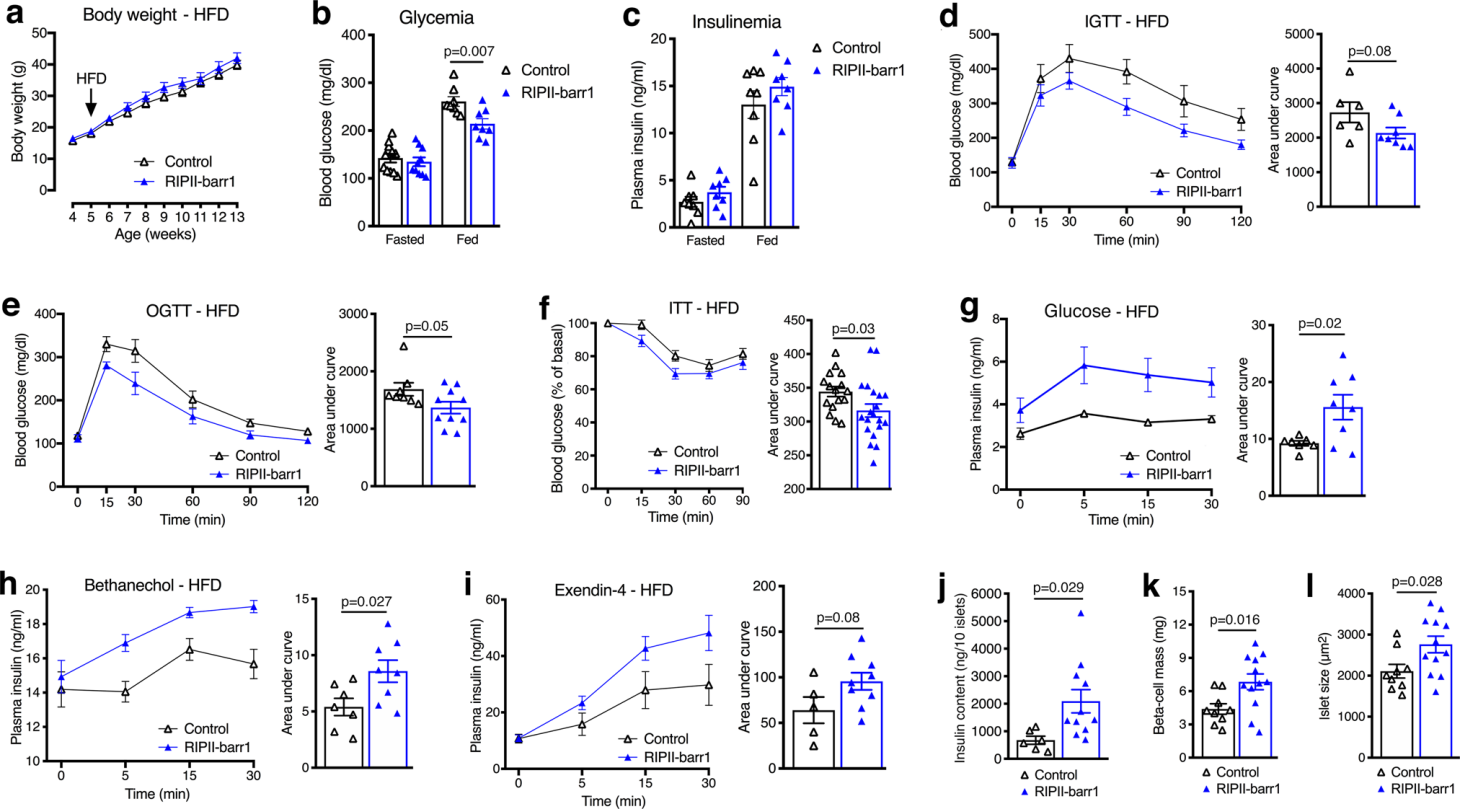
Mice overexpressing barr1 in beta-cells (RIPII-barr1 mice) exhibit improved glucose homeostasis on an obesogenic diet. For all experiments, adult male RIPII-barr1 mice and WT littermates maintained on a high-fat diet (HFD) for at least 8 weeks were used. **a** Body weight gain of RIPII-barr1 mice and WT littermates maintained on a HFD. **b**, **c** Blood glucose (**b**) and plasma insulin (**c**) levels of freely fed or fasted (14–16 h overnight) mice. Measurements were performed with male littermates that had consumed the HFD for at least 8 weeks. **d** I.p. glucose tolerance test (IGTT). After an overnight fast, mice were injected with glucose (1 g/kg i.p.). **e** Oral glucose tolerance test (OGTT). After an overnight fast, mice received glucose via oral gavage (1 g/kg i.p.). **f** Insulin tolerance test (ITT). Mice were fasted for 4 h and then injected with insulin (1 U/kg i.p.). **g** Glucose-stimulated increases in plasma insulin levels. Mice that had been fasted overnight were injected with glucose (1 g/kg i.p.), followed by the monitoring of plasma insulin levels. **h** Bethanechol-stimulated insulin secretion. Freely fed mice were injected with bethanechol (2 mg/kg s.c.). **i** Exendin-4-stimulated insulin secretion. Mice that had been fasted for 4 h were injected with exendin-4 (12 nmoles/kg i.p.) in combination with glucose (1 g/kg i.p.). **j** Insulin content of pancreatic islets (*n* = 6–11 batches of ten islets from at least three different mice per genotype were analyzed). **k**, **l** Morphometric analysis of beta-cell mass (**k**) and islet size (**l**) in HFD control and RIPII-barr1 mice (three slices from three or four mice/genotype were analyzed). Data were given as means ± s.e.m. (number of mice used: **a**, 8 per group; **b**, 10 for fasted and 8 for fed mice, respectively; **c**, 8 per group; **d**, 6 control and 8 for RIPII-barr1 mice, respectively; **e**, 8 control and 10 for RIPII-barr1 mice, respectively; **f**, 16 control and 20 RIPII-barr1 mice, respectively; **g**, **h**, 7 control and 8 RIPII-barr1 mice, respectively; **i**, 5 control and 9 RIPII-barr1 mice, respectively; **j**, 6 control and 11 RIPII-barr1 mice, respectively; **k**, **l**, 9 control and 12 RIPII-barr1 mice, respectively (mouse age: 15–22 weeks). *P* values are indicated in the different panels (unpaired two-tailed *t*-test). Source data are provided as a Source data file.

To test whether enhanced expression of barr1 in beta-cells might ameliorate the metabolic deficits associated with obesity, we generated a transgenic mouse line that overexpressed barr1 in beta-cells under the transcriptional control of the rat insulin promoter II (RIPII-barr1 mice; Supplementary Fig. 9a). *Barr2* expression levels remained unaffected in RIPII-barr1 mice, as compared to WT littermates (Supplementary Fig. 9b). When maintained on standard chow, RIPII-barr1 mice did not display any significant metabolic phenotypes, except for a small decrease in plasma insulin levels in the fed state (Supplementary Fig. 9c–h). In contrast, RIPII-barr1 mice that had consumed a HFD for at least 8 weeks showed metabolic phenotypes that were opposite to those observed with the HFD beta-barr1-KO mice (Fig. 7). While HFD WT and RIPII-barr1 mice showed similar body weight (Fig. 7a), the transgenic mice exhibited a significant decrease in blood glucose levels (fed state; Fig. 7b), and improved glucose tolerance (IGTT and OGTT; Fig. 7d, e) and insulin sensitivity (ITT; Fig. 7f). Moreover, in the RIPII-barr1 mice, plasma insulin levels were greatly increased in response to glucose (Fig. 7g), bethanechol (Fig. 7h), and exendin-4 (Fig. 7i). In striking contrast to our findings with HFD beta-barr1-KO mice, islet insulin content (Fig. 7j), beta-cell mass (Fig. 7k), and mean islet size (Fig. 7l) were significantly increased in HFD RIPII-barr1 mice. These data support the concept that beta-cell barr1 plays a key role in the compensatory increase in beta-cell mass that is triggered by peripheral insulin resistance.

## Discussion

It is well known that obese individuals display impaired peripheral insulin sensitivity (insulin resistance)^1–3^, which, in most cases, triggers a compensatory increase in beta-cell mass. This increase in beta-cell mass is able to delay or even prevent the development of T2D^4–7^. For this reason, therapeutic approaches aimed at enhancing beta-cell mass are considered to be of great potential clinical benefit.

In the present study, we identified barr1 as a factor/intracellular protein that is essential for the expansion of beta-cell mass resulting from peripheral insulin resistance induced by an obesogenic diet. Specifically, we demonstrated that mice that selectively lacked barr1 in beta-cells (beta-barr1-KO mice) showed pronounced reductions in beta-cell mass and the rate of beta-cell proliferation when maintained on a calorie-rich HFD (Fig. 2a–e). As a result, HFD beta-barr1-KO mice showed severely impaired glucose homeostasis, including impaired glucose tolerance, reduced GSIS, and increased blood glucose levels (Fig. 1). Most strikingly, the absence of beta-cell barr1 led to a pronounced reduction in Pdx1 transcript and protein levels in HFD beta-barr1-KO mice (Figs. 2f and 3a, b). We obtained similar findings after knockdown of barr1/BARR1 expression in mouse and human beta-cell lines (Figs. 4a, b and 5).

We also noted that *Ngn3* and *Snap25* transcript levels were significantly increased in HFD beta-barr1-KO islets (Fig. 2f). Ngn3 is a transcription factor that is critical for the development of the endocrine cells of the pancreatic islets^56^, and enhanced *Ngn3* expression may lead to an increase in beta-cell mass and improved beta-cell function^56^. The Snap25 protein is a component of the trans-SNARE complex, which plays an essential role in stimulus-dependent release of insulin from beta-cells^57^. These findings raise the possibility that the observed increases in *Ngn3* and *Snap25* expression represent compensatory responses caused by the reduced expression of *Pdx1* and other functionally critical beta-cell genes (Fig. 2f).

In adult beta-cells, Pdx1 represents the master regulator of beta-cell function and the proper regulation of beta-cell mass^25,26^. Consistent with the finding that Pdx1 is required for the proper expression of many functionally critical beta-cell genes, including *MafA*, *Ins2*, and *Glut2*^41,42^, the expression of *MafA*, *Ins2*, and *Glut2* was reduced in HFD beta-barr1-KO islets (Fig. 2f). Thus, it is likely that the altered expression levels of these latter genes are secondary to the decrease in *Pdx1* expression. Previous studies have shown that reduced Pdx1 expression in heterozygous *Pdx1* mutant mice prevents the increase in beta-cell mass caused by peripheral insulin resistance^43,44^. In agreement with this finding, humans heterozygous for an inactivating mutation of *PDX1* suffer from maturity-onset diabetes of the young (MODY 4)^58^. Taken together, these findings suggest that the metabolic deficits displayed by HFD beta-barr1-KO mice were primarily due to decreased Pdx1 expression levels.

While *Pdx1* mRNA expression was reduced by ~50% in HFD beta-barr1-KO islets (Fig. 2f), Pdx1 protein expression levels were decreased to an even greater extent (by ~80%; Fig. 3a, b), suggesting that barr1 may also play a role in stabilizing Pdx1. The very pronounced reduction of Pdx1 protein in HFD beta-barr1-KO islets may explain why heterozygous *Pdx1* mutant mice in which Pdx1 protein expression is reduced by 50% do not show impairments in glucose homeostasis until later in life^43,59^.

Previous studies have shown that the stability of Pdx1 protein is regulated by the activity of multiple signaling proteins including AKT and GSK-3β^60^. It is well known that β-arrestins regulate pathways that involve AKT and GSK-3β signaling in various cell types^19^. Thus, one possibility is that the pronounced reduction in Pdx1 protein expression caused by barr1 deficiency is an indirect effect due to altered activity of certain intracellular signaling cascades. The precise nature of the signaling proteins involved in this process remains to be elucidated.

To further corroborate the concept that reduced Pdx1 expression is the primary cause of the impaired beta-cell function observed with HFD beta-barr1-KO islets (Fig. 1j), we treated HFD beta-barr1-KO islets with an adenovirus coding for Pdx1 (Ad-Pdx1). We found that Ad-Pdx1-treated mutant islets regained a similar magnitude of GSIS as control islets treated with Ad-Pdx1 or a control adenovirus (Ad-GFP) (Fig. 3c). This rescue experiment further supports the notion that Pdx1 deficiency resulting from the lack of beta-cell barr1 is the key factor responsible for the impairment in beta-cell function displayed by beta-barr1-KO islets.

Because of the potential therapeutic relevance of our findings, we carried out a series of additional studies with EndoC-βH1 cells, a human beta-cell line that closely mimics the functional features of native human beta-cells46,47. Similar to the results obtained with HFD beta-barr1-KO islets (Figs. 1j and 2f), treatment of EndoC-βH1 with *BARR1* siRNA cells led to severe deficits in GSIS and PDX1 expression (Figs. 4 and 5). The observation that insulin content was unchanged in *BARR1*-deficient EndoC-βH1 cells but decreased in HFD beta-barr1-KO islets, may be due to the fact that barr1 expression was reduced chronically (for weeks or months) in the islets, while insulin content of EndoC-βH1 cells was measured 2 days after *BARR1* siRNA treatment.

Studies with EndoC-βH1 cells also showed that the expression levels of AKT, FOXO1, CREB, ERK, and GSK3β, as well as the expression levels of the phosphorylated forms of these signaling proteins/transcription factors, remained essentially unchanged after *BARR1* knockdown (Fig. 5), suggesting that BARR1 deficiency does not interfere with the important roles of these proteins in maintaining proper beta-cell function. This observation again suggests that reduced PDX1 expression levels, in conjunction with the decreased expression of PDX1-regulated genes (Fig. 4b) are most likely responsible for the metabolic impairments observed with BARR1-deficient EndoC-βH1 cells. However, since the ability of bethanechol and exendin-4 to stimulate insulin release was impaired in HFD beta-barr1-KO mice (Fig. 1), we cannot exclude the possibility that these agents affect the phosphorylation status of downstream signaling molecules after *BARR1* knockdown in EndoC-βH1 cells.

Studies with HFD beta-barr1-KO islets as well as *BARR1* siRNA-treated EndoC-βH1 cells showed that KCl-induced insulin secretion remained unaffected by beta-cell barr1 (BARR1) deficiency (Figs. 1k and 4c). This finding suggests that lack of beta-cell barr1 does not interfere with distal events of the insulin exocytosis machinery.

Previous studies have shown that nuclear barr1 can modulate gene expression at the transcriptional level^15–18^. Studies with a reporter construct suitable for measuring mouse *Pdx1* promoter activity demonstrated that nuclear barr1 is required for efficient activation of the mouse *Pdx1* promoter (Fig. 6a). Consistent with this finding, ChIP experiments identified a mouse *Pdx1* promoter region that was protected by both anti-p300 and anti-barr1 antibodies (Fig. 6c, d). Since barr1 can directly interact with p300^15^, this observation suggests that p300 and barr1 are part of a protein complex that enhances *Pdx1* transcription. At present, it remains unclear which upstream signaling molecules or pathways promote the formation of complexes containing barr1 and Pdx1. This important topic will be the subject of future investigations.

RNA-seq studies with islet RNA prepared from HFD beta-barr1-KO and control islets demonstrated that beta-cell barr1 deficiency led to reduced expression levels of genes involved in cell cycle progression including several genes coding for different cyclins and cyclin-pendent kinases (Fig. 3d). Interestingly, a recent study^61^ showed that Pdx1 overexpression stimulates beta-cell proliferation and that this effect is linked to the upregulation of several cell cycle genes. These observations establish a link between downregulation of Pdx1 expression and impaired beta-cell proliferation found with HFD beta-barr1-KO mice.

HFD RIPII-barr1 mice that overexpressed barr1 selectively in beta-cells showed metabolic phenotypes that were opposite to those observed with the HFD beta-barr1-KO mice (Fig. 7). This finding supports the concept that barr1 plays a key role in regulating beta-cell function and proliferation under obesogenic conditions. However, it remains to be explored whether the metabolic improvements displayed by HFD RIPII-barr1 mice involve molecular pathways that are similar to those affected by beta-cell barr1 deficiency. Without additional experimentation, we cannot rule out the possibility that alternative pathways are responsible for the metabolic changes caused by overexpression of barr1 in beta-cells.

We recently demonstrated that *barr1* expression tended to be reduced in islets prepared from mice maintained on a HFD, as compared to islets from mice consuming regular chow^10^. Moreover, human islets cultured for several days under glucolipotoxic conditions showed a significant reduction (by ~40%) in *BARR1* expression^10^. Taken together, these published data, together with the current data obtained with human EndoC-βH1 cells in the present study, strongly suggest that beta-cell BARR1 may also play a critical role in human beta-cell dysfunction under pathophysiological conditions.

In conclusion, we provide evidence that beta-cell barr1 is required for the adaptive increase in beta-cell mass in obesity and proper beta-cell function in the insulin-resistant state. Moreover, we identified the molecular mechanism that links barr1 to the expression of *Pdx1* and other beta-cell genes that are critical for beta-cell proliferation and function. Our findings suggest that drugs able to increase the expression or activity of beta-cell barr1 may prove beneficial for the treatment or prevention of T2D.

## Methods

### Drugs, reagents, commercial kits, and antibodies

All drugs, reagents, commercial kits, and antibodies and their sources are listed in Supplementary Table 1.

### Animals and mouse maintenance

Beta-barr1-KO mice (tamoxifen-treated *barr1 fl/fl Pdx1-Cre-ER*^*TM*^ mice) and control littermates (*barr1 fl/fl* mice) were generated by standard techniques^24^. To induce Cre activity in beta-cells of adult mice, 8-week-old *barr1 fl/fl Pdx1-Cre-ER*^*TM*^ and *barr1 fl/fl* mice were injected i.p. with tamoxifen once a day for 5 consecutive days (2 mg/day/mouse, dissolved in sterile corn oil). Two weeks after the last tamoxifen injection, mice were switched from regular chow to a HFD. Mice were maintained on the HFD for at least 8 weeks before initiating metabolic studies. To generate mice overexpressing barr1 in beta-cells, we constructed a transgene in which the expression of an HA-tagged version of rat barr1 (Addgene) was under the transcriptional control of the rat insulin promoter II (RIPII)^62^. The linearized construct was microinjected into the pronuclei of fertilized ova from C57BL/6NTac mice (Taconic, Germantown, NY) using standard transgenic techniques. Mice harboring the *RIPII-barr1* transgene were identified via PCR of genomic tail DNA. To detect the *RIPII-barr1* transgene, we used the following PCR primers (size of PCR product, 415 bp): forward, 5′-ATTTGAGGGACGCTGTG-3′; reverse, 5′-TTGATGAGTCGCTCTTGTAG-3′. PCR cycling conditions were as follows: 95 °C for 5 min, followed by 35 cycles at 95 °C for 30 s, 55 °C for 30 s, and 72 °C for 1 min. We identified a mouse line that selectively overexpressed barr1 in pancreatic islets (see text for details). Throughout the text, we refer to these mice simply as RIPII-barr1 mice. Hemizygous RIPII-barr1 mice were crossed with WT C57BL/6 NTac mice, and WT littermates served as control animals. All mice were maintained on a pure C57BL/6NTac background. C57BL/6NTac mice are derived from C57BL/6N mice which were distributed to Taconic by the NIH Animal Genetic Resource in 1991 (https://www.taconic.com/mouse-model/black-6-b6ntac).

We complied with all relevant ethical regulations for animal testing and research. All animal studies were approved by the National Institute of Diabetes and Digestive and Kidney Diseases/NIH Animal Care and Use Committee. Mice were fed ad libitum, kept on a 12-h light/dark cycle, and maintained at room temperature (23 °C). Mice consumed a standard chow (7022 NIH-07 diet, 15% kcal fat, energy density 3.1 kcal/g, Envigo Inc.) or a HFD (F3282, 60% kcal fat, energy density 5.5 kcal/g, Bioserv) for at least 8 weeks, unless stated otherwise.

### Studies with MIN6-K8 cells

MIN6-K8 mouse insulinoma cells (source: Dr. Susumu Seino, Kobe University, Japan) were cultured in Dulbecco’s modified Eagle’s medium (DMEM) containing 10% heat-inactivated fetal bovine serum and maintained in a humidified incubator with 95% air and 5% CO2 at 37 °C. For *barr1* knockdown studies, ~1.5 × 10^6^ cells were electroporated using the Nucleofector^TM^ kit (VCA-1002, Lonza) and 100 nM of mouse *barr1* siRNA or scrambled control siRNA (siGenome SMARTpool siRNA, a pool  of four different siRNAs; cat. # M-040976-01-0005; Horizon/Dharmacon), according to the manufacturer’s instructions. For studies involving the Pdx1-pGL3 luciferase reporter construct^50^, cells were co-transfected with siRNA (either *barr1* or control siRNA) and the Pdx1-pGL3 plasmid or empty pGL3 vector. Cells were harvested 48 h after electroporation, and luciferase activity was assessed using a Luciferase Assay System (E1501, Promega) and a multi-mode plate reader (SpectraMax M5, Molecular Devices).

### Studies with human EndoC-βH1 beta-cells

Human EndoC-βH cells were a kind gift by Dr. Raphael Scharfmann (INSERM U1016, Institut Cochin, Universite Paris Descartes, France)^49^. EndoC-βH1 cells were cultured in a humidified incubator with 95% air and 5% CO2 at 37 °C with DMEM containing 1 g/l glucose, 2% albumin from bovine serum fraction V (Equitech), 50 μM 2-mercaptoethanol (Sigma), 10 mM nicotinamide (Millipore), 5.5 μg/ml transferrin (Sigma), 6.7 ng/ml sodium selenite (Sigma), and penicillin (100 units/ml)/streptomycin (100 μg/ml). The culture flasks were pre-coated with medium containing 1% ECM (Sigma) and 2 μg/ml fibronectin (Sigma). For gene silencing studies, ~1.5 × 10^6^ cells were electroporated with 100 nM of human *BARR1* siRNA or scrambled control siRNA (ON-TARGETplus siRNA; Horizon Discovery). Cells were harvested 48 h after electroporation and stored at −80 °C until RNA extraction (see below) or lysed and directly used for Western blotting studies. Following treatment with *BARR1* or control siRNA, EndoC-βH1 cells were incubated with a low or high concentration of glucose (1 and 20 mM, respectively) to measure GSIS. Specifically, cells were preincubated in Krebs–Ringer buffer (NaCl 115 mM; NaHCO_3_ 24 mM; KCl 5 mM; MgCl_2_ 1 mM; CaCl_2_ 1 mM; HEPES 10 mM; BSA 0.2%) containing 1 mM glucose for 1 h. After this preincubation step, the supernatant was removed and cells were incubated with Krebs–Ringer buffer containing 1 mM glucose, 20 mM glucose, or 30 mM KCl. After 60 min, the supernatant was collected and stored at −20 °C. Insulin concentrations in the samples were measured using a commercial ELISA kit for human insulin (Crystal Chem).

### In vivo metabolic studies

I.p. and oral glucose tolerance test (IGTT and OGTT, respectively) were carried out using standard techniques. After an overnight (14–16 h) fast, mice received glucose either via i.p. injection or via oral gavage (1 g glucose/kg). Blood glucose levels were measured from the tail vein before and at defined time points after glucose administration using a Bayer Contour glucometer. For insulin tolerance tests (ITT), mice were fasted for 4 h (10 a.m. to 2 p.m.) and then injected with human insulin (1 U/kg i.p., Humulin R, Eli Lilly). Blood glucose levels were measured before and at specific time points after insulin injection. To stimulate insulin release in vivo, mice were injected with glucose (1 g/kg i.p.), exendin-4 (12 nmoles/kg i.p.), or bethanechol (2 mg/kg s.c.). Plasma insulin and glucagon levels were determined using commercial ELISA kits (Crystal Chem and Mercodia, respectively).

### Perifusion studies with isolated islets

Mouse pancreatic islets were isolated by using a published protocol^63^. An automated perifusion system was utilized to dynamically measure insulin secretion (Biorep Perifusion System, Miami, FL). A peristaltic pump pushed a HEPES-buffered solution (composition in mM: 125 NaCl, 5.9 KCl, 2.56 CaCl2, 1.2 MgCl2, 25 HEPES, and 0.1% BSA [pH 7.4]; perifusion rate: 100 μl/min) through a sample container with the islets immobilized in Bio-Gel P-4 Gel (Bio-Rad, Hercules, CA). Islets were preincubated with 3 mM glucose for 1 h and then incubated with 16 mM glucose for 15 min, followed by a final 20 min incubation step with 3 mM glucose. Glucose was applied with the HEPES-buffered solution, and the perfusate was collected every minute in a 96-well plate for further analysis. The chambers containing the islets were kept at 37 °C, and the perfusate in the collecting plate was kept at <4 °C. Insulin concentrations in the perfusates were determined with an ultrasensitive mouse insulin ELISA kit (Mercodia, Winston Salem, NC).

### Static insulin secretion studies with isolated mouse islets

Mouse pancreatic islets from HFD control and HFD beta-barr1-KO mice were isolated by standard techniques^63^. Batches of isolated islets (~50 islets per batch) were infected with adenoviruses coding for GFP (control) or Pdx1 (Vector Biolabs, ADV-268353) for 24 h at 50 MOI. After this step, batches of ten islets were incubated for 1 h at 37 °C in Krebs–Ringer bicarbonate/HEPES buffer containing 2.8 mM glucose, followed by another 1 h incubation step at 28 mM glucose. The amount of insulin secreted into the medium during each 1 h incubation period was normalized to the total insulin content of the islets in each well^63^. Insulin levels were determined using a commercial ELISA kit (Crystal Chem).

### Western blotting

Immunoblotting studies were performed using standard techniques^63^. Primary antibodies were diluted 1:1000 or 1:2000 (beta-actin). Secondary antibodies were diluted 1:3000 (for antibody details, see Supplementary Table 1). Immunoreactive bands were detected using an Azure C600 gel imaging system (Azure Biosciences; Dublin, CA) and quantified using ImageJ software (National Institutes of Health, Bethesda, MD).

### Preparation of nuclear and cytoplasmic protein extracts

After treatment of EndoC-βH1 cells with *BARR1* or scrambled control siRNA (see “Studies with human EndoC-βH1 beta-cells”), cells were subjected to a nuclear/cytoplasmic protein fractionation protocol (Nuclear Extraction Kit; Abcam, ab113474), following the manufacturer’s instructions. Nuclear and cytoplasmic protein extracts were then used for Western blotting studies to determine BARR1 localization. Lamin A/C and beta-tubulin were used as nuclear and cytoplasmic marker proteins, respectively.

### Morphometric studies of pancreatic islets

Briefly, mouse pancreata were fixed overnight in 4% paraformaldehyde/phosphate-buffered saline, followed by embedding in paraffin. Pancreatic sections (5 μm thick) from three distinct levels (100 μm apart) were mounted on slides and subjected to standard hematoxylin/eosin staining. To determine beta-cell mass and islet size, three distinct sections per pancreas (100 μm apart) were blocked with normal goat serum for 1 h and incubated overnight at 4 °C with a guinea pig anti-insulin antibody and a rabbit anti-glucagon antibody. Additional slides were used for detecting pancreatic delta-cells, using a rabbit anti-somatostatin antibody. The insulin and glucagon antibodies were detected with Alexa Fluor 555 goat anti-guinea pig (red color) and Alexa Fluor 488 goat anti-rabbit (green color) secondary antibodies, respectively. The somatostatin antibody was detected with Alexa Fluor 488 goat anti-rabbit secondary antibody (green color). All sections were counterstained with DAPI (Vectashield mounting medium with DAPI, Vector Laboratories) to visualize the nuclei (blue color). Slides were imaged using a Keyence digital microscope (BZ-9000) with a CFI Plan Apo l 4x lens. Image acquisition and merging were performed using BZ-II Viewer and BZ-II Analyzer software (Keyence). ImageJ was used for the measurement of beta-, alpha-, and delta-cell mass, which were calculated as the ratio of islet cross-sectional area to total pancreatic area multiplied by pancreatic weight. Islet size was calculated by dividing islet area by the total number of islets. Beta-cell size was measured by imaging randomly selected beta-cells at 400X. Beta-cell size was determined as mean individual beta-cell cross-sectional area^64^. Islets from three mice per genotype were analyzed (75 beta-cells per islet; 12 islets per mouse).

### Measurement of beta-cell proliferation and apoptosis

Beta-cell proliferation was measured via insulin and Ki67 co-staining of pancreatic slices prepared from beta-barr1 KO and control littermates that had been maintained on a HFD for 12 weeks. Ki67-insulin double-positive cells were counted and divided by the total number of insulin-positive cells per pancreatic section, presented as percentage. Three pancreatic sections were analyzed per mouse.

To study beta-cell apoptosis, we subjected pancreatic slices from control and beta-barr1-KO maintained on a HFD for 8 weeks to TUNEL staining using the DeadEnd Fluorometric TUNEL System, according to the manufacturer’s (Promega) instructions. Beta-cells were identified by an anti-insulin antibody.

### Quantification of RNA expression via qRT-PCR

RNA was extracted from pancreatic islets and various other mouse tissues utilizing the RNeasy Mini Kit (Qiagen, Germantown, MD), following the manufacturer’s instructions. cDNA was prepared using Superscript III first-strand synthesis SuperMix for qRT-PCR (Thermo Scientific). PCR reactions were run in triplicate using SYBR Green Master Mix (Applied Biosystems, Foster City, CA) in a real-time PCR detection system (Bio-Rad, Hercules, CA). Primer sequences are given in Supplementary Table 2. Relative quantification of gene expression was done by using the 2^−ΔΔCt^ method (difference between the threshold cycle (Ct) value of the target gene and the Ct of the beta-actin housekeeping gene).

### RNA-seq studies

Total RNA was extracted from isolated pancreatic islets of beta-barr1-KO and their control littermates that had been maintained on a HFD for ~10 weeks after the induction of the barr1 knockout. RNAs were tested for quality using a Bioanalyzer Instrument (Agilent, Santa Clara, CA). RIN >8 was the threshold used for determination of RNA quality. High-throughput sequencing was performed using a HiSeq 2500 Sequencing System (Illumina). The mouse genome mm9 was used to map the raw data. The Genomatix genome analyzer was employed to identify differentially expressed genes. Enrichment analysis and the analysis of biological pathways were performed using Metacore (Clarivate Analytics), Ingenuity Pathway Analysis (Qiagen), and Partek Flow (Partek). For the generation of heatmaps, functional gene enrichment analyses were performed using ToppGene Suite (http://toppgene.cchmc.org). Genes enriched in selected GO pathways were visualized as heatmaps using Partek Flow. The RNA-seq data can be downloaded from the NCBI Sequence Read Archive under reference number RNA seq-PRJNA578926.

### Pull-down assay

Barr1 pull-down assays were performed with lysates prepared from MIN6-K8 cells using a Dynabeads^TM^ Co-Immunoprecipitation kit (Thermo Scientific), following the manufacturer’s instructions. Briefly, anti-barr1 antibody (Cell Signaling, # 12697) or normal rabbit IgG (Cell Signaling, # 2729) (2 μg of antibody or IgG per 1 mg of beads) were covalently coupled to the epoxy beads provided by the kit. Beads were then incubated for 30 min at 4 °C with lysates (250 μg protein per sample) prepared from MIN6-K8 cells. Beads were then washed, and proteins were eluted from the beads with elution buffer containing LDS sample buffer (Thermo Scientific) and analyzed by Western blotting employing the same anti-barr1 antibody used for the immunoprecipitation step.

### Chromatin immunoprecipitation (ChIP) assay

MIN6-K8 cells were transfected with either *barr1* or scrambled control siRNA via electroporation. Two days after transfection, cells were fixed with formaldehyde, and ChIP assays were carried out using the Abcam ChIP kit (ab500), following the manufacturer’s instructions. Antibodies against barr1 and p300 were used to immunoprecipitate barr1- and p300-bound chromatin sequences. Subsequently, qPCR was performed on the eluted DNA using multiple primer pairs in order to amplify distinct regions within the *Pdx1*, *Mafa*, and *Cdkn3* promoters predicted to bind p300-regulated transcription factors (AliBaba2.1; http://gene-regulation.com/pub/programs/alibaba2/index.html).

### Electron microscopic analysis of mouse beta-cells

HFD beta-barr1-KO and control mice were anesthetized with tribromoethanol (250 mg/kg i.p) and then transcardially perfused with PBS for 30 s followed by a fixative solution (2.5% glutaraldehyde, 0.5% tannic acid, and 30 mM sucrose in 0.1 M cacodylate buffer) for 5 min. The pancreas was then removed and postfixed for 2 h in the same fixative solution, followed by 1 h in 1% osmium tetroxide in 0.1 M cacodylate buffer. Tissue was trimmed in small blocks of about 1 mm^3^, washed three times in buffer, and then stained en bloc with 2% uranyl acetate in 50% ethanol for 1 h, followed by dehydration and embedding in Epon-Araldite resin. Subsequently, blocks were cut in 60 nm ultrathin sections, stained with lead citrate, and imaged in a FEI Technai T12 transmission electron microscope at 1900x magnification. Images were collected from at least three different blocks and three different islets per animal. Image analysis was performed with the help of ImageJ software.

### Statistical analysis

Data were expressed as means ± s.e.m. Mean values between two groups were compared using an unpaired *t*-test (two-tailed) or two-way ANOVA followed by Sidak’s post hoc test. *P* values < 0.05 were considered statistically significant. The specific statistical tests that were used are indicated in the figure legends.

### Study approval

All animal studies were approved by the NIDDK Institutional Animal Care and Use Committee (NIH, Bethesda, MD).

### Reporting Summary

Further information on research design is available in the Nature Research Reporting Summary linked to this article.

## Supplementary information

Supplementary Information

Reporting Summary

## Data Availability

All the other data supporting the findings of this study are available within the article and its supplementary information files and from the corresponding author upon reasonable request. A reporting summary for this article is available as a Supplementary Information file. The RNA-seq data can be downloaded from the NCBI Sequence Read Archive under reference number RNA seq-PRJNA578926. Source data are provided with this paper.

## Source data

Source Data
