# Supplementary material for: β-Arrestin-1 is required for adaptive β-cell mass expansion during obesity
Source: Nat Commun. 2021 Jun 7;12:3385. doi: 10.1038/s41467-021-23656-1 (PMC8184739; doi:10.1038/s41467-021-23656-1)
Supplement: Supplementary file 1 — Supplementary Information [file 41467_2021_23656_MOESM1_ESM.pdf]

Supplemental Information

β-Arrestin-1 is Required for Adaptive β-Cell Mass Expansion During Obesity

Luiz F. Barella[1], Mario Rossi[1], Sai P. Pydi[1], Jaroslawna Meister[1], Shanu Jain[2], Yinghong Cui[1], Oksana Gavrilova[3], Gianluca Fulgenzi[4], Lino Tessarollo[4], and Jürgen Wess[1]

[1]Molecular Signaling Section, Laboratory of Bioorganic Chemistry, National Institute of Diabetes and Digestive and Kidney Diseases, Bethesda, MD 20892, USA
[2]Molecular Recognition Section, Laboratory of Bioorganic Chemistry, National Institute of Diabetes and Digestive and Kidney Diseases, Bethesda, MD 20892, USA
[3]Mouse Metabolism Core, National Institute of Diabetes and Digestive and Kidney Diseases, Bethesda, Bethesda, MD 20892, USA
[4]Mouse Cancer Genetics Program, National Cancer Institute, Frederick 21702, MD, USA

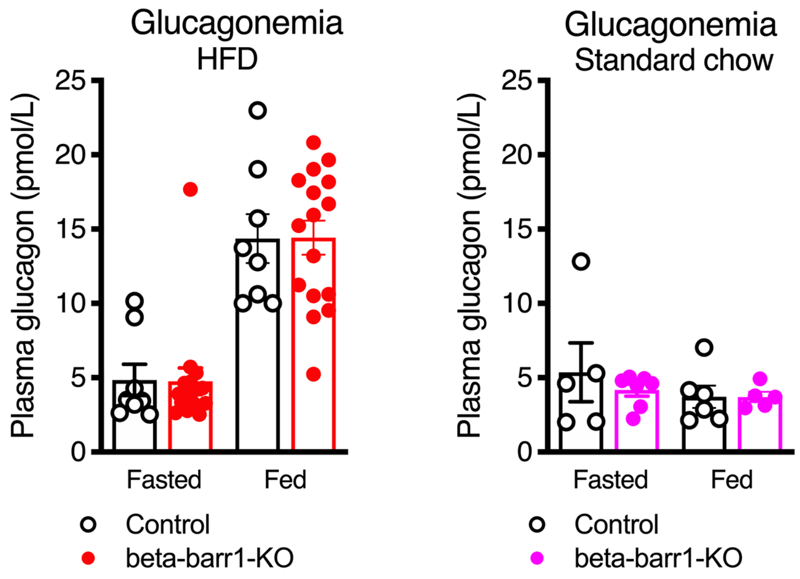

Supplementary Fig. 1. Plasma glucagon levels are similar in HFD beta-barr1-KO mice and control littermates. Plasma glucagon levels were determined in fasted (14-16 hr overnight) or freely fed mice. Mice were maintained either on a HFD or standard chow. Data are given as means ± s.e.m. (left panel: 8 control and 16 KO mice under both fasting and fed conditions; right panel: 5 control and 7 KO mice under fasting conditions; 6 control and 5 KO mice under fed conditions). Mouse age: 19-20 weeks (left panel) and 11-12 weeks (right panel) respectively. Source data are provided as a Source data file.

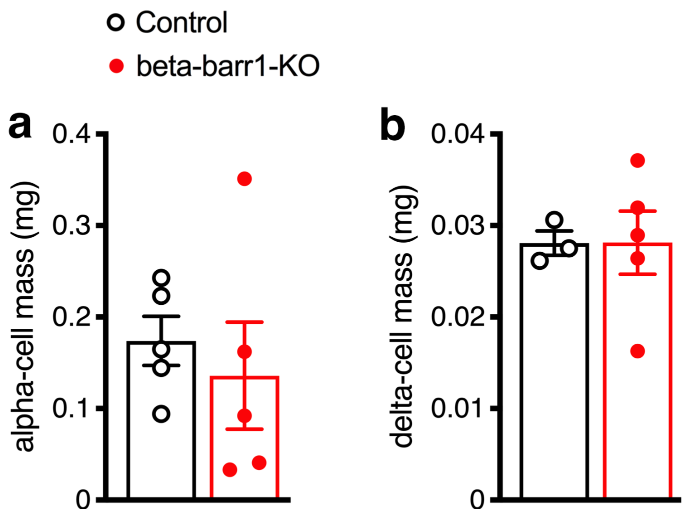

Supplementary Fig. 2 Alpha-cell and delta-cell mass remained unchanged by beta-cell barr1 deficiency. **a, b** Alpha-cell mass and (**a**) and delta-cell mass (**b**) are similar in HFD beta-barr1-KO and HFD control mice. Data are given as means ± s.e.m. (a, 5 control and 5 KO pancreatic sections were examined from 3 mice per genotype; b, 3 control and 5 KO pancreatic sections were examined from 3 mice per genotype; mouse age: 18 weeks). Source data are provided as a Source data file.

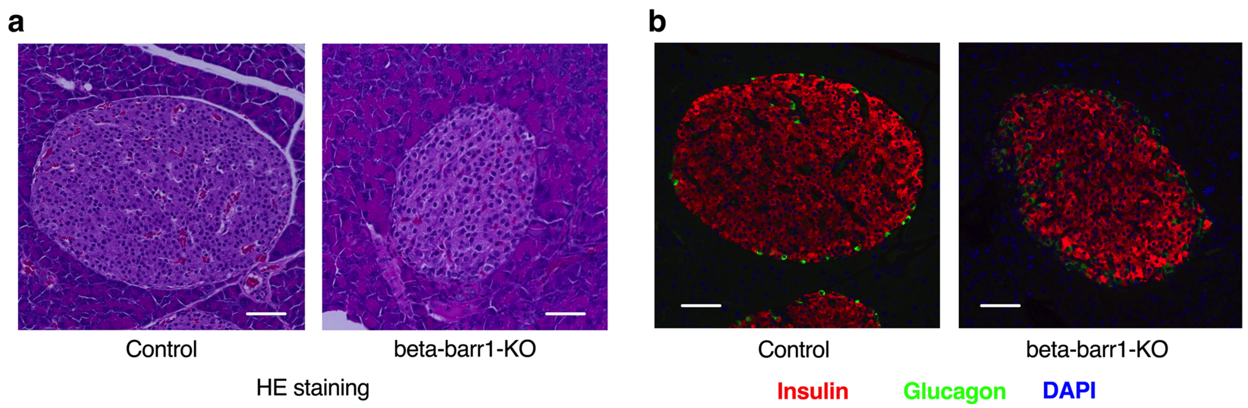

Supplementary Fig. 3. Overall islet architecture is similar in HFD beta-barr1-KO mice and control littermates. a, b Representative images of islets from HFD beta-barr1-KO mice and HFD control littermates stained with H&E (**a**) or with anti-insulin and anti-glucagon antibodies (**b**). Pancreatic sections were analyzed from 5 mice per group. Scale bar, 100 μm.

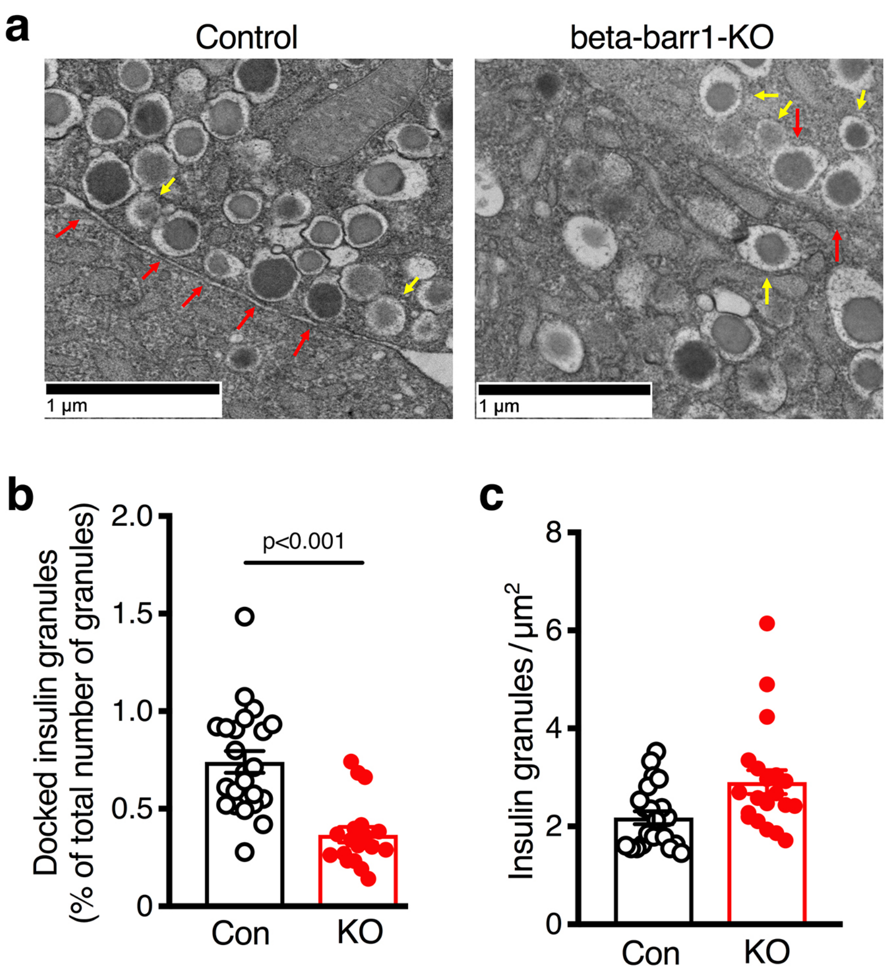

Supplementary Fig. 4. The number of pre-docked insulin granules is reduced in beta-cells lacking barr1. a Representative transmission electron microscope images of beta-cells from control (Con) and beta-barr1-KO (KO) mice (4800x magnification). Insulin vesicles pre-docked to the plasma membrane are indicated by red arrows, while not-docked vesicles are indicated by yellow arrows. Note that the docked vesicles frequently show an increase in electron dense material between the plasma and the vesicle membrane. Scale bar, 1 µm. **b** Quantification of pre-docked insulin granules as percentage of total number of granules, normalized by µm of membrane. **c** Quantification of the total number of insulin granules per μm^2. Data are given as means ± s.e.m. (n=20-24 beta-cells from 3 mice per group). *P* value is indicated in panel **b** (unpaired two-tailed t-test). Source data are provided as a Source data file.

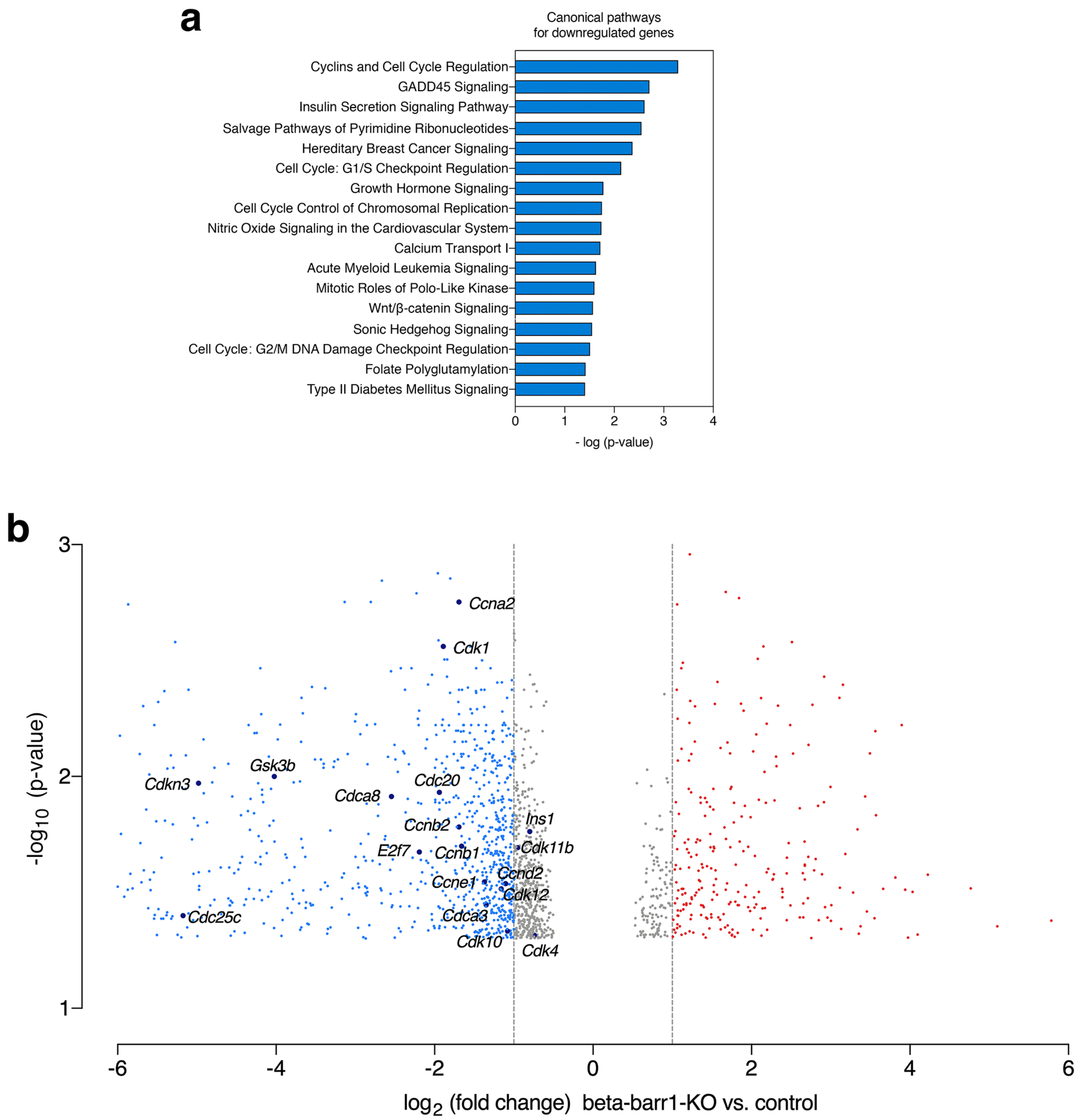

Supplementary Fig. 5. RNA-seq studies with RNA from islets of HFD beta-barr1-KO mice and control littermates. RNA-seq analysis was used to determine changes in gene expression in pancreatic islets prepared from HFD beta-barr1-KO mice vs. HFD control littermates. **a** Summary of most significantly enriched signaling pathways for significantly downregulated genes. **b** Volcano plot with downregulated genes shown in blue, upregulated genes in red, and unchanged genes in gray. A total of 263 genes were upregulated, whereas 841 genes were downregulated (fold change > 2). Differentially expressed genes (DEGs) were identified ($p<0.05$) by using EdgeR in Genomatix Genome Analyzer. DEGs are expressed as $\log_2$ fold change over control with an adjusted p value for each gene. Key genes are highlighted. Islet RNA was collected from male littermates that had been maintained on a HFD for ~10 weeks (n=6 mice per genotype; age: ~20 weeks).

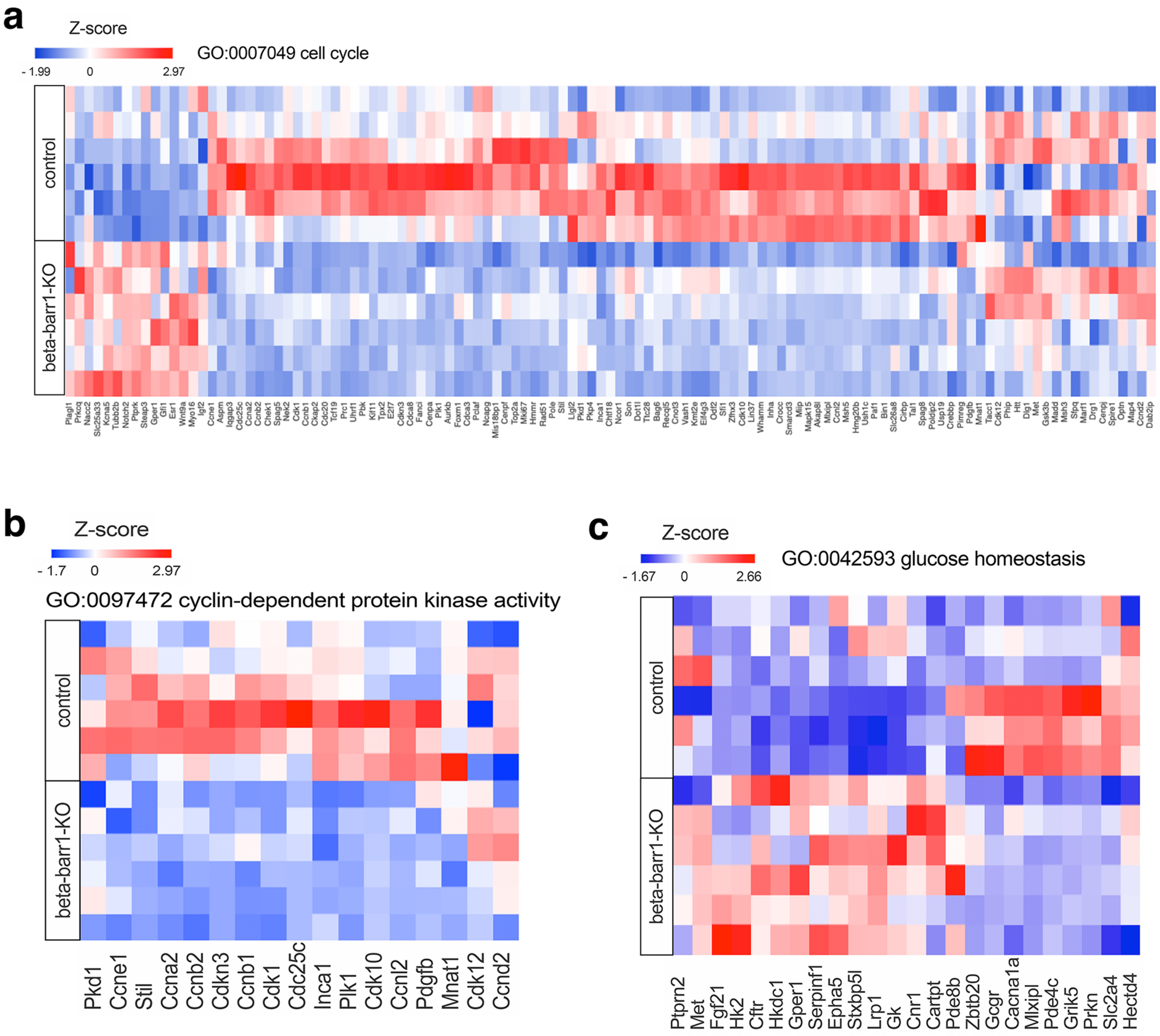

Supplementary Fig. 6. Heat maps displaying color-coded expression levels of differentially expressed genes. RNA-seq analysis was used to determine changes in gene expression in pancreatic islets prepared from HFD beta-barr1-KO mice and HFD control littermates. **a-c** Heat map displays of differentially expressed genes (cut-off: log2(1) or 2-fold). The heat maps show the differential expression of genes involved in cell cycle (GO term: 0007049; 116 out of a total of 1971 genes) (**a**), cyclin-dependent protein kinase activity (GO term: 0097472; 17 out of a total of 133 genes) (**b**), and glucose homeostasis (GO term: 0042593; 24 out of a total of 294 genes (**c**), generated using Partek Flow. Islet RNA was collected from male littermates that had been maintained on a HFD for ~10 weeks (n=6 mice per genotype; age: ~20 weeks).

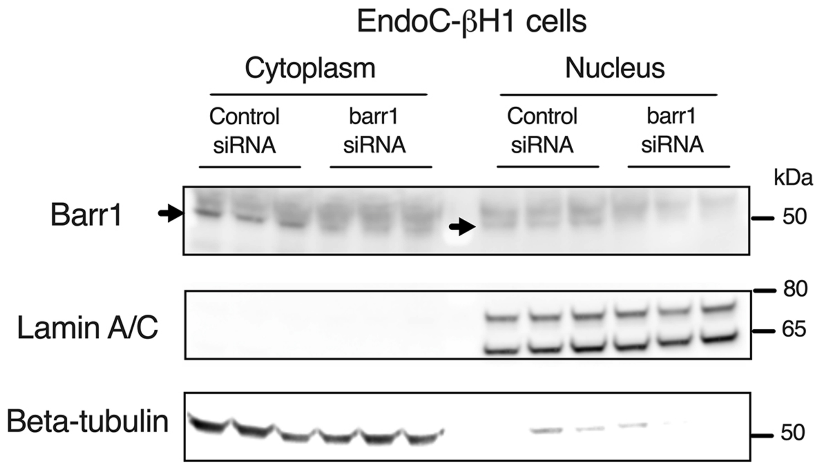

Supplementary Fig. 7. Human EndoC-βH1 cells express endogenous Barr1 in nuclear and cytosolic protein extracts, as determined by Western blotting. Treatment of cells with *BARR1* siRNA significantly decreases Barr1 expression in both fractions. Lamin A/C and beta-tubulin were used as nuclear and cytoplasmic marker proteins, respectively. The blots shown are representative of 3 independent experiments.

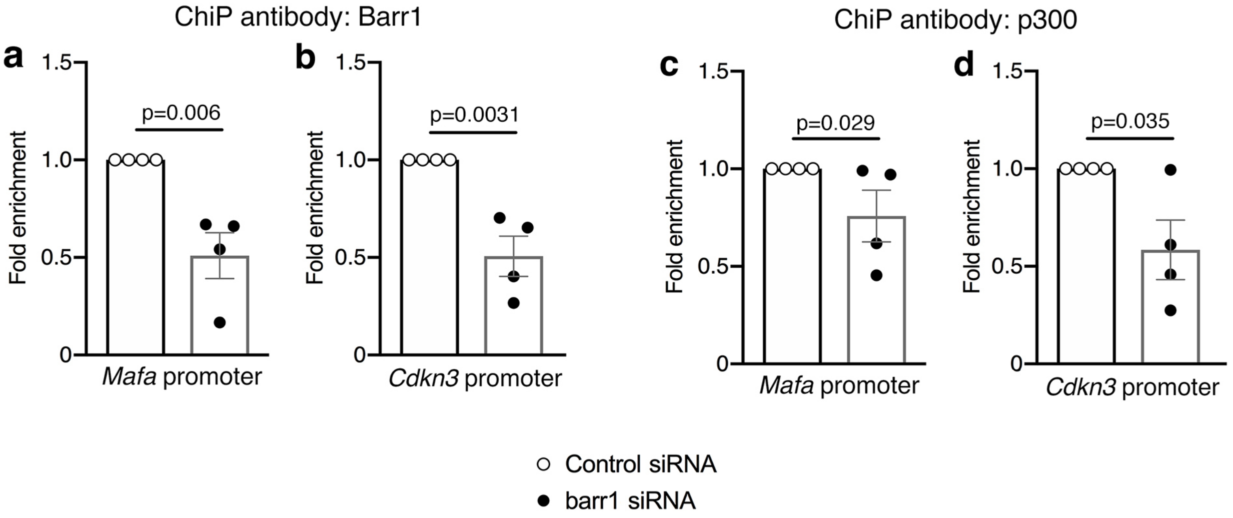

Supplementary Fig. 8. Beta-cell barr1 deficiency leads to decreased fold enrichment at the *MafA* and *Cdkn3* promoters in MIN6-K8 cells. a-d ChIP experiments were carried out using antibodies against barr1 and p300 with MIN6-K8 cells transfected with either scrambled control or *barr1* siRNA. The presence of *MafA* and *Cdkn3* promoter sequences in the input DNA recovered from the antibody-bound chromatin segments was analyzed by qPCR. Data were normalized to the corresponding input controls. Primers used for qPCR studies are listed in Supplementary Table 2. Data are presented as means ± s.e.m. from four independent ChIP experiments. *P* values are indicated in the different panels (unpaired two-tailed t-test). Source data are provided as a Source data file.

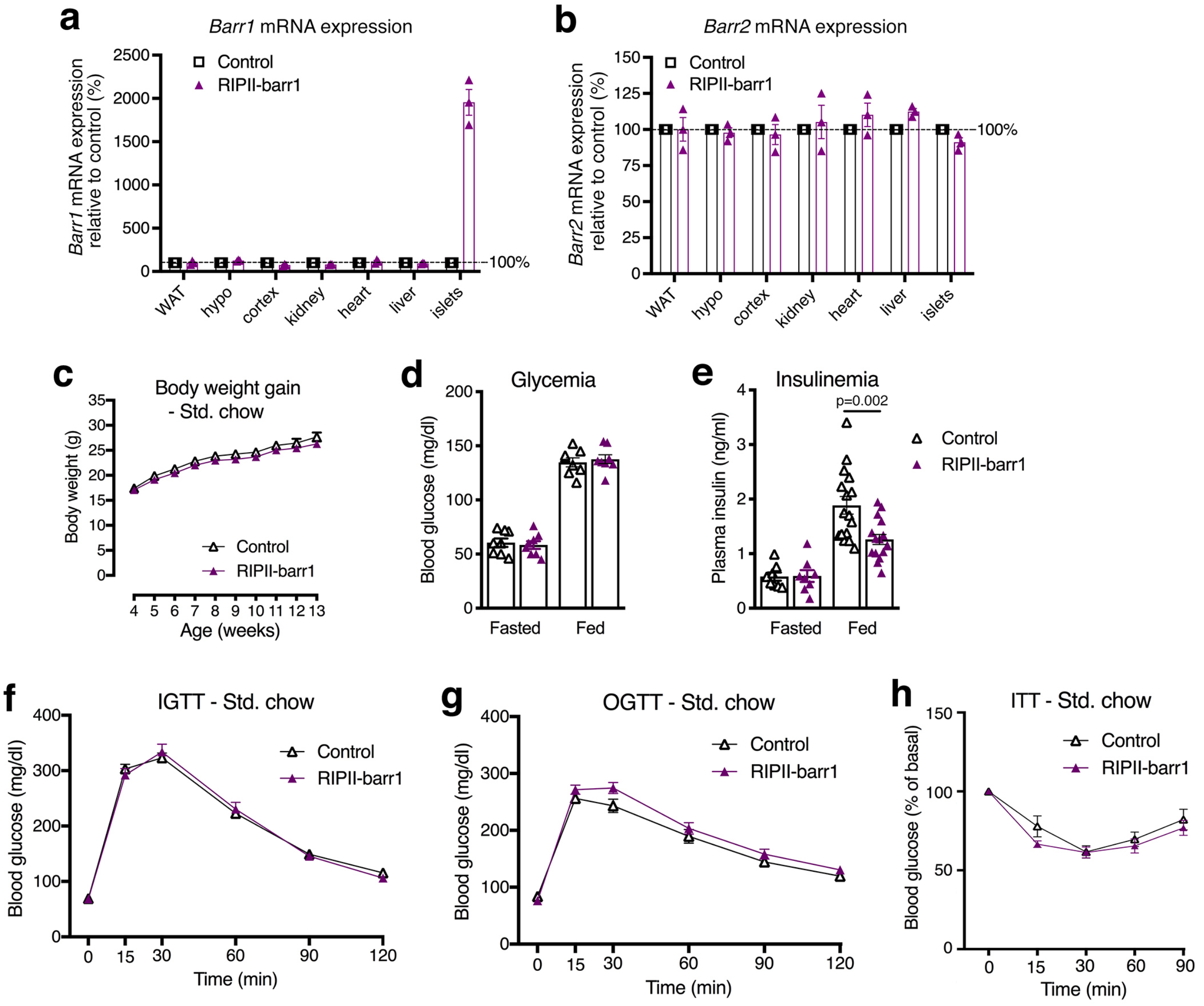

Supplementary Fig. 9. Mice overexpressing barr1 in beta-cells (RIPII-barr1 mice) show unchanged glucose tolerance and insulin sensitivity when maintained on standard chow. All experiments were carried out with male RIPII-barr1 mice and WT control littermates maintained on standard mouse chow. (**a, b**) qRT-PCR studies indicating selective overexpression of *barr1* in pancreatic islets of RIPII-barr1 mice (A). *Barr2* mRNA levels were similar in tissues from control (WT) and RIPII-barr1 mice (B). WAT, white adipose tissue; hypo, hypothalamus; cortex, cerebral cortex. (**c**) Body weight measurements. (**d, e**) Blood glucose (d) and plasma insulin (e) levels in freely fed or fasted (14-16 hr overnight fast) mice. (**f**) I.p. glucose tolerance test (IGTT). After an overnight fast, mice were injected with glucose (2 g/kg i.p.). (**g**) Oral glucose tolerance test (OGTT). After an overnight fast, mice received an oral gavage of glucose (2 g/kg i.p.). (**h**) Insulin tolerance test (ITT). Mice were fasted for 4 hr and then injected with insulin (1 U/kg i.p.). Blood glucose levels were measured at the indicated time points.
Data are given as means ± s.e.m. (number of mice per group: a and b, 3; c and d, 8; e, 8 for fasted and 16 for fed mice, respectively; f, 8; g, 10 control and 9 RIPII-barr1 mice, respectively; h, 8) (mouse age: 12-16 weeks). *P* value is indicated in panel **e** (unpaired two-tailed t-test). Source data are provided as a Source data file.

Supplementary Table 1. Source of reagents and animals

REAGENT or RESOURCE	SOURCE	IDENTIFIER
**Antibodies**		
Pdx1	Cell Signaling	5679S
p300	Cell Signaling	86377
β-Arrestin-1	Cell Signaling	12697
β-Actin	Cell Signaling	8457
p-Akt (T308)	Cell Signaling	2975
p-Akt (S473)	Cell Signaling	9271
Akt	Cell Signaling	9272
p-Foxo1	Cell Signaling	9461
Foxo1	Cell Signaling	14952
p-Creb	Cell Signaling	9198
Creb	Cell Signaling	9197
p-Erk1/2 (p44/42 MAPK)	Cell Signaling	4376
Erk1/2 (p44/42 MAPK)	Cell Signaling	9102
p-Gsk3β	Cell Signaling	5558
Gsk3β	Cell Signaling	9315
Irs-2	Cell Signaling	3089
Lamin A/C	Cell Signaling	4777
β-tubulin	Cell Signaling	2146
Anti-rabbit IgG, HRP-linked secondary antibody	Cell Signaling	7074S
Anti-mouse IgG, HRP-liked secondary antibody	Cell Signaling	7076S
Normal rabbit IgG	Cell Signaling	2729
Ki67	Abcam	ab15580
Insulin (guinea pig)	Abcam	ab7842
Glucagon (rabbit)	ThermoFisher	RB-1422-A1
Somatostatin (rabbit)	Abcam	Ab108456
Alexa Fluor 555 goat anti-guinea pig	ThermoFisher	A21435
Alexa Fluor 488 goat anti-rabbit	ThermoFisher	A11034
**Chemicals, Peptides, and Recombinant Proteins**		
BCA protein assay	Pierce	23225
ECL western blotting substrate	Pierce	32106
Bovine serum albumin (fatty acid-free)	Sigma-Aldrich	A7030
Nucleofector™ kit	Lonza	VCA-1002
Tween 20	Fisher Scientific	BP337
TRIzol	ThermoFisher	15596026
RNase-free DNase I	Qiagen	79254
Power SYBR Green PCR Master Mix	Applied Biosystems	4367659
RNeasy mini kit	Qiagen	74104
SuperScript™ III First-Strand Synthesis SuperMix	Invitrogen	18080400
cOmplete EDTA-free protease inhibitor cocktail	Sigma-Aldrich	11873580001
NuPAGE LDS sample buffer	Thermo Fisher Scientific	NP0007
**Critical Commercial Assays**		
Rat insulin ELISA kit	Crystal Chem	90010

Mouse insulin standard	Crystal Chem	90020
Human insulin ELISA kit	Crystal Chem	90095
Mouse glucagon ELISA kit	Mercodia	10-1281-01
Ultrasensitive mouse insulin ELISA kit	Mercodia	10-1249-01
ChIP kit	Abcam	ab500
Dynabeads Co-Immunoprecipitation kit	Thermo Fisher Scientific	14321D
Nuclear Extraction kit	Abcam	Ab113474
**Experimental Models: Cell Lines**		
MIN6-K8 cells	Gift by Dr. Susumu Seino[1]	
EndoC-βH1 cells	Provided by Dr. Raphael Scharfmann[2]	
**Experimental Models: Organisms/Strains**		
Pdx1-Cre-ER^{TM} mice	Provided by Dr. Doug Melton[3]	
RIPII-barr1 mice	Generated at the NIDDK for this study	
Barr1 f/f mice	Provided by Dr. Robert Lefkowitz[4]	
**Recombinant DNA**		
Pdx1-pGL3 construct	Provided by Dr. Anabel Rojas[5]	
pGL3 luciferase reporter vector	Promega	E1751
**Software and Algorithms**		
Prism 7	Graph Pad	https://www.graphpad.com/scientific-software/prism/
ImageJ	ImageJ	http://imagej.net/Adiposoft
Metacore	Clarivate Analytics	https://portal.genego.com/
Ingenuity Pathway Analysis	Qiagen	https://www.qiagenbioinformatics.com/products/ingenuity-pathway-analysis/
Partek Flow	Partek	https://www.partek.com/partek-flow/

Supplementary Table 2. Primers used for qRT-PCR studies

**Mouse gene**	**Primer sequence**	**Amplicon (bp)**
Beta-actin	QuantiTect Primer (Qiagen)<br>Catalog #: QT01136772	77
Barr1 (Arrb1)	Forward: 5' AAGAAGGCAAGCCCCAAT<br>Reverse: 5' CGCAGGTCAGTGTCACGTAG	151
Barr2 (Arrb2)	Forward: 5' GTCTTCAAGAAGTCGAGCCCT<br>Reverse: 5' CACGAACACTTTCCGGTCCT	144
Cav1.2	QuantiTect Primer (Qiagen)<br>Catalog #: QT00150752	115
Cav1.3	QuantiTect Primer (Qiagen)<br>Catalog #: QT00112238	96
Ins2	Forward: 5' CTGGCCCTGCTCTTCCTCTGG<br>Reverse: 5' CTGAAGGTCACCTGCTCCCGG	204
Irs2	Forward: 5' CTGCGTCCTCTCCCAAAGTG<br>Reverse: 5' GGGGTCATGGGCATGTAGC	124
Pdx1	Forward: 5' CCCCAGTTTACAAGCTCGCT<br>Reverse: 5' CTCGGTTCCATTCGGGAAAGG	177
MafA	Forward: 5' AGGAGGAGGTCATCCGACTG<br>Reverse: 5' CTTCTCGCTCTCCAGAATGTG	113
Gck	Forward: 5' AGGAGGCCAGTGTAAAGATGT<br>Reverse: 5' CTCCCAGGTCTAAGGAGAGAAA	90
Sur1 (Abcc8)	QuantiTect Primer (Qiagen)<br>Catalog #: QT01042300	77
Kir6.2 (Kcnj11)	QuantiTect Primer (Qiagen)<br>Catalog #: QT00305319	87
Pcx	Forward: 5' CTGAAGTTCCAAACAGTTCGAGG<br>Reverse: 5' CGCACGAAACACTCGGATG	162
Glut2	Forward: 5' TCAGAAGACAAGATCACCGGA<br>Reverse: 5' GCTGGTGTGACTGTAAGTGGG	215
Ucp2	Forward: 5' ATGGTTGGTTTCAAGGCCACA<br>Reverse: 5' TTGGCGGTATCCAGAGGGAA	113
Nkx6.1	Forward: 5' CAGCAAATCTTCGCCCTGGA<br>Reverse: 5' AACCAGACCTTGACCTGACT	116
Snap25	Forward: 5' CAACTGGAACGCATTGAGGAA<br>Reverse: 5' GGCCACTACTCCATCCTGATTAT	177
Stx1a	Forward: 5' GAGCCAGGGGGAGATGATTGA	194

	Reverse: 5' ATCCAAAGATGCCCCCGATG	
Vamp2	Forward: 5' GCTGGATGACCGTGCAGAT<br>Reverse: 5' GATGGCGCAGATCACTCCC	130
Epac2	Forward: 5' CAAGATGTCTTGGTACTGGAGAAG<br>Reverse: 5' CAGGTGTTCCTGACATCACAGTAT	103
Ngn3	Forward: 5' ACGCAATTTACTCCAGGCGA<br>Reverse: 5' GAGGCGCCATCCTAGTTCTC	169
NeuroD1	Forward: 5' ACCTTTTAACAACAGGAAGTGGA<br>Reverse: 5' CTCATCTGTCCAGCTTGGGG	101
Cdk2	Forward: 5' CAAAGCCAAGCACGTAGAGAC<br>Reverse: 5' TGCACCACATATTGACTGTCC	141
Pdx1 promoter	Forward: 5' TGGAGGACCAGGTCAGAGG<br>Reverse: 5' GCGCTGGCAAGGATAGACT	114
Mafa promoter	Forward: 5' CTGGAACCTCAGAATCTGCCA<br>Reverse: 5' GCCCAGCTGTCAATCTCCTG	150
Cdkn3 promoter	Forward: 5' AAAGCCAGCTAAATGAAGACTGAA<br>Reverse: 5' GCGCAAGGAACTGAACTAGG	125
**Human gene**	**Primer sequence**	**Amplicon (bp)**
PDX1	Forward: 5' GGAGCTGGCTGTCATGTTG<br>Reverse: 5' CACTTCATGCGGCGGTTT	72
MAFA	Forward: 5' GAGAGCGAGAAGTGCCAACT<br>Reverse: 5' CTTGTACAGGTCCCGCTCTTT	87
INS	Forward: 5' AGGCCATCAAGCAGATCACT<br>Reverse: 5' TGTTGGTTCACAAAGGCTGC	117
BARR1 (ARRB1)	Forward: 5' TCAAGCACGAAGACACGAAC<br>Reverse: 5' ATGCAAGATCTCCCAACAGG	141
BARR2 (ARRB2)	Forward: 5' CACGTCACCAACAACTCCAC<br>Reverse: 5' TTGTTCGAGTTGAGCCACAG	120
HPRT	Forward: 5' TTGCTTTCCTTGGTCAGGCA<br>Reverse: 5' ATCCAACACTTCGTGGGGTC	85

1 Iwasaki, M. *et al.* Establishment of new clonal pancreatic beta-cell lines (MIN6-K) useful for study of incretin/cyclic adenosine monophosphate signaling. *Journal of diabetes investigation* **1**, 137-142, doi:10.1111/j.2040-1124.2010.00026.x (2010).

2 Scharfmann, R. *et al.* Development of a conditionally immortalized human pancreatic beta cell line. *J Clin Invest* **124**, 2087-2098, doi:10.1172/jci72674 (2014).

3 Gu, G., Dubauskaite, J. & Melton, D. A. Direct evidence for the pancreatic lineage: NGN3+ cells are islet progenitors and are distinct from duct progenitors. *Development (Cambridge, England)* **129**, 2447-2457 (2002).

4 Kim, J. *et al.* beta-arrestin 1 regulates beta2-adrenergic receptor-mediated skeletal muscle hypertrophy and contractility. *Skeletal muscle* **8**, 39, doi:10.1186/s13395-018-0184-8 (2018).

5 Carrasco, M., Delgado, I., Soria, B., Martin, F. & Rojas, A. GATA4 and GATA6 control mouse pancreas organogenesis. *J Clin Invest* **122**, 3504-3515, doi:10.1172/jci63240 (2012).
