# Supplementary material for: β-Arrestin-1 is required for adaptive β-cell mass expansion during obesity
Source: Nat Commun. 2021 Jun 7;12:3385. doi: 10.1038/s41467-021-23656-1 (PMC8184739; doi:10.1038/s41467-021-23656-1)
Supplement: Supplementary file 2 — Reporting Summary [file 41467_2021_23656_MOESM2_ESM.pdf]

nature research

Corresponding author(s): Jürgen Wess

Last updated by author(s): 04-24-2021

Reporting Summary

Nature Research wishes to improve the reproducibility of the work that we publish. This form provides structure for consistency and transparency in reporting. For further information on Nature Research policies, see Authors & Referees and the Editorial Policy Checklist .

Statistics

For all statistical analyses, confirm that the following items are present in the figure legend, table legend, main text, or Methods section.

n/a	Confirmed	
☐	☒	The exact sample size (*n*) for each experimental group/condition, given as a discrete number and unit of measurement
☐	☒	A statement on whether measurements were taken from distinct samples or whether the same sample was measured repeatedly
☐	☒	The statistical test(s) used AND whether they are one- or two-sided<br>*Only common tests should be described solely by name; describe more complex techniques in the Methods section.*
☐	☒	A description of all covariates tested
☐	☒	A description of any assumptions or corrections, such as tests of normality and adjustment for multiple comparisons
☐	☒	A full description of the statistical parameters including central tendency (e.g. means) or other basic estimates (e.g. regression coefficient) AND variation (e.g. standard deviation) or associated estimates of uncertainty (e.g. confidence intervals)
☐	☒	For null hypothesis testing, the test statistic (e.g. *F*, *t*, *r*) with confidence intervals, effect sizes, degrees of freedom and *P* value noted<br>*Give P values as exact values whenever suitable.*
☒	☐	For Bayesian analysis, information on the choice of priors and Markov chain Monte Carlo settings
☒	☐	For hierarchical and complex designs, identification of the appropriate level for tests and full reporting of outcomes
☒	☐	Estimates of effect sizes (e.g. Cohen's *d*, Pearson's *r*), indicating how they were calculated

Our web collection on statistics for biologists contains articles on many of the points above.

Software and code

Policy information about availability of computer code

Data collection	Prism 7, Excel (version 16), ImageJ (version 2.1.0), Metacore, Ingenuity Pathway Analysis (version 01-16), Partek Flow (version 10)
Data analysis	Prism 7, Excel (version 16), ImageJ (version 2.1.0), Metacore, Ingenuity Pathway Analysis (version 01-16), Partek Flow (version 10)

For manuscripts utilizing custom algorithms or software that are central to the research but not yet described in published literature, software must be made available to editors/reviewers. We strongly encourage code deposition in a community repository (e.g. GitHub). See the Nature Research guidelines for submitting code & software for further information.

Data

Policy information about availability of data

All manuscripts must include a data availability statement. This statement should provide the following information, where applicable:

- Accession codes, unique identifiers, or web links for publicly available datasets
- A list of figures that have associated raw data
- A description of any restrictions on data availability

Data availability
All the other data supporting the findings of this study are available within the article and its supplementary information files and from the corresponding author upon reasonable request. A reporting summary for this article is available as a Supplementary Information file. The RNA-seq data can be downloaded from the NCBI Sequence Read Archive under reference number RNA seq-PRJNA578926. Source data are provided with this paper.

Field-specific reporting

Please select the one below that is the best fit for your research. If you are not sure, read the appropriate sections before making your selection.

☒ Life sciences ☐ Behavioural & social sciences ☐ Ecological, evolutionary & environmental sciences

For a reference copy of the document with all sections, see nature.com/documents/nr-reporting-summary-flat.pdf

Life sciences study design

All studies must disclose on these points even when the disclosure is negative.

Sample size	Sample size was chosen based on prior experience of the investigators with similar experiments previously published. The authors have published numerous peer-reviewed papers demonstrating clear positive findings with similar sample sizes for the types of experiments included.
Data exclusions	No data points were excluded from the analysis of any of the experiments.
Replication	All experimental findings were reproduced in at least three independent experiments.
Randomization	Randomization was performed by blinding investigators to genotype and allowing them to choose each subject blindly.
Blinding	Randomization was performed by blinding investigators to genotype and allowing them to choose each subject blindly. For studies using pharmacological agents, the investigator was aware of the agent being used, but was not aware of the genotypes of the animals used. Investigators were not aware of the specific group to which an animal was assigned to when doing the experiment or until after completion of the experiment.

Reporting for specific materials, systems and methods

We require information from authors about some types of materials, experimental systems and methods used in many studies. Here, indicate whether each material, system or method listed is relevant to your study. If you are not sure if a list item applies to your research, read the appropriate section before selecting a response.

Materials & experimental systems

n/a	Involved in the study	
☐	☒	Antibodies
☐	☒	Eukaryotic cell lines
☒	☐	Palaeontology
☐	☒	Animals and other organisms
☒	☐	Human research participants
☒	☐	Clinical data

Methods

n/a	Involved in the study	
☒	☐	ChIP-seq
☒	☐	Flow cytometry
☒	☐	MRI-based neuroimaging

Antibodies

Antibodies used

All antibodies were used at 1:1000 dilution, except fir the following:
Beta-actin: 1:2000
Anti-rabbit IgG: 1:3000
Anti-mouse IgG: 1:3000
Insulin (guinea pig), Glucagon (rabbit): 1:100
Somatostatin (rabbit): 1:200
Alexa Fluor 555 and 488: 1:500
Ki67: 1:500

For CoIP: 2ug of antibody per sample
For ChIP: 5ug pf antibody per sample

Pdx1 Cell Signaling 5679S
p300 Cell Signaling 86377
Beta-arrestin-1 Cell Signaling 12697 Beta-Actin Cell Signaling 8457
p-Akt (T308) Cell Signaling 2975
p-Akt (S473) Cell Signaling 9271

	Akt Cell Signaling 9272<br>p-Foxo1 Cell Signaling 9461<br>Foxo1 Cell Signaling 14952<br>p-Creb Cell Signaling 9198<br>Creb Cell Signaling 9197<br>p-Erk1/2 (p44/42 MAPK) Cell Signaling 4376 Erk1/2 (p44/42 MAPK) Cell Signaling 9102 p-Gsk3beta Cell Signaling 5558<br>Gsk3beta Cell Signaling 9315<br>Irs-2 Cell Signaling 3089<br>Lamin A/C Cell Signaling 4777-tubulin<br>Cell Signaling 2146<br>Anti-rabbit IgG, HRP-linked secondary antibody Cell Signaling 7074S Anti-mouse IgG, HRP-liked secondary antibody Cell Signaling 7076S Normal rabbit IgG Cell Signaling 2729<br>Ki67 Abcam ab15580<br>Insulin (guinea pig) Abcam ab7842<br>Glucagon (rabbit) ThermoFisher RB-1422-A1<br>Somatostatin (rabbit) Abcam Ab108456<br>Alexa Fluor 555 goat anti-guinea pig ThermoFisher A21435
Validation	Validated by the manufacturer via immunohistochemistry and Western blotting

Eukaryotic cell lines

Policy information about cell lines

Cell line source(s)	MIN6-K8 cells (gift by Dr. Susumu Seino)<br>EndoC-betaH1 cells (Provided by Dr. Raphael Scharfmann)
Authentication	Authenticated in our lab by insulin secretion studies
Mycoplasma contamination	The cell lines tested negative for mycoplasma contamination.
Commonly misidentified lines<br>(See ICLAC register)	No commonly misidentified cell lines were used in the study.

Animals and other organisms

Policy information about studies involving animals; ARRIVE guidelines recommended for reporting animal research

Laboratory animals	Male mice (C57BL6 background) older than 8 weeks; Housing conditions: Room temperature 23 oC; humidity, 40%
Wild animals	No wild animals were used in the study.
Field-collected samples	No field collected samples were used in the study.
Ethics oversight	All animal studies were approved by the NIDDK institutional Animal Care and Use Committee.

Note that full information on the approval of the study protocol must also be provided in the manuscript.
